# The antibiotic procurement saga: a long-neglected stewardship target to combat antimicrobial resistance in Pakistan

**DOI:** 10.1186/s13756-025-01521-w

**Published:** 2025-02-07

**Authors:** Shairyar Afzal, Mishal Bajwa, Nabeel Ahmed, Jawaria Jabeen, Mian Shahzeb Haroon, Rana Muhammad Zahid Mushtaq, Zikria Saleem

**Affiliations:** 1 Department of Pharmacy Practice, Faculty of Pharmacy, Hamdard University, Islamabad Campus, Islamabad, Pakistan; 2 Department of Pharmacy, DHQ Hospital Jhelum, Jhelum, Pakistan; 3 Department of Pharmacy, Quaid-i-Azam University, Islamabad, 45320 Pakistan; 4 College of Pharmacy, University of Sargodha, Sargodha, Pakistan; 5 Department of Pharmacology and Toxicology, University of Veterinary and Animal Sciences, Lahore, Pakistan; 6 Institute for Regeneration and Repair (IRR), Edinburgh Medical School, The University of Edinburgh, Edinburgh, UK; 7 Faculty of Pharmacy, Bahauddin Zakariya University, Multan, Punjab 60800 Pakistan

**Keywords:** Antibiotic procurement, Antimicrobial stewardship program (ASP), Antimicrobial resistance (AMR), Antibiotic access, Antibiotic use policy, Clinical pharmacist

## Abstract

**Background:**

Consistent and timely access to antibiotics is a hallmark of an antimicrobial stewardship program (ASP) and can be achieved through good procurement practices. However, flawed procurement modules result in poor antibiotic supply management within health facilities of low- and middle-income countries (LMICs), including Pakistan, exacerbating antimicrobial resistance (AMR). This study seeks to understand hospital pharmacists’ perspectives on the antibiotic procurement process, its efficiency in ensuring consistent access to antibiotics, and the role of clinical pharmacists in rational procurement.

**Methods:**

Semi-structured interviews with 24 purposively selected hospital pharmacists from secondary healthcare facilities in Punjab, Pakistan, were conducted utilizing a qualitative case study methodology. Data analysis was conducted using MAXQDA 2024 software, following a thematic analysis technique using a codebook approach to thematic analysis.

**Results:**

The study identified five central themes: (1) The state of antibiotic use in hospitals is characterized by a lack of antibiotic use policy, resulting in mostly empirical and irrational prescribing practices. (2) Medicine availability significantly influences prescribing decisions, often taking precedence over clinical needs. (3) The procurement process, although structured, is flawed owing to rigid adherence to the Standard Medicine List (SML). (4) Rationality in procurement is compromised by disregard for AMR, with decisions driven more by cost and demand than clinical evidence. (5) The clinical acumen of pharmacists is underutilized in procurement due to multifarious barriers.

**Conclusion:**

Antibiotic procurement is the mainstay of implementing an ASP in hospitals. This study elucidates significant policy, practice, and education gaps regarding antibiotic use and procurement in Pakistan. There is a critical need for comprehensive antibiotic policies, including a revision in SML, enhancing pharmacist authority in procurement decisions, more rational prescribing, and ensuring access to antibiotics through more informed and data-driven processes to combat AMR effectively.

**Supplementary Information:**

The online version contains supplementary material available at 10.1186/s13756-025-01521-w.

## Background

The United Nations Sustainable Development Goal (SDG) target 3.8 entails access to safe, effective, quality, and affordable essential medicines for all [1]. Holistically, access to medicine is influenced by the complex interplay of multi-pronged factors, including regulatory matters (intellectual property licences and registration), financial stability (government’s affordability), research and development (market entry rewards and economic models for antimicrobials), healthcare system (infrastructure), collaboration and cooperation (inter-country and/or private organizations), socio-economic factors, supply chain mechanism, pricing policies, procurement strategies and appropriate use (particularly for antimicrobials) [2–11]. Consistent access to antibiotics offers the necessary restraint against antimicrobial resistance (AMR) and is guaranteed by efficient procurement practices [3, 12]. However, irrational use and inefficient procurement mechanisms in low- and middle-income countries (LMICs), including Pakistan, have resulted in insufficient and inequitable access to antibiotics [13], significantly accelerating the problem of AMR and further compromising the already weakened stewardship infrastructure [14, 15].

A well-designed procurement module based on an evidence-based medicine list guided by an antibiotic use policy, which considers local epidemiology and resistance patterns, along with added guidance from the WHO AWaRe system, will ensure optimal antibiotic management within the health facility [16]. The antimicrobial stewardship program (ASP), which aims to optimize antibiotic therapy and limit AMR, warrants timely availability of the appropriate antibiotic, which is a cornerstone of an effective treatment strategy and is highly dependent on efficient procurement practices and subsequent inventory control within health facilities. Despite this, antibiotic procurement has been given negligible importance as an essential component of stewardship programs [17, 18].

The World Health Organization (WHO) espoused the Global Action Plan (GAP) on AMR in 2015, advocating equitable access to antibiotics while simultaneously optimizing their use through stewardship interventions [19]. In response to the GAP’s call, Pakistan promulgated its National Action Plan (NAP) in May 2017 to combat AMR using the former as a blueprint [20]. However, NAP Pakistan faces implementation challenges owing to its inability to address localized context-specific details [21]. Furthermore, NAP Pakistan is unable to address possible strategies to cope with the fragile supply chain system and ensure consistent access to antibiotics throughout health facilities. Several previous studies have reported antibiotic shortages and reduced availability, particularly in public hospitals in Pakistan, but were unable to examine the effect of the procurement process and factors thereof responsible for the shortage or unavailability of antibiotics within these healthcare setups [22–24]. Addressing this gap is essential for developing effective AMR control strategies by implementing this critical component of ASP in LMICs, including Pakistan [25]. This study explores underlying factors determining consistent access to antibiotics through hospital pharmacists’ perspectives on the antibiotic procurement process and its current status as a stewardship tool to restrain AMR within secondary healthcare facilities. This study is also interested in investigating whether the procurement process can be rationalized by directly engaging a clinical pharmacist or by employing a pharmacist’s clinical input.

## Methods

### Study setting

Post 18th Constitutional Amendment, health is steered by Provincial governments [26]. The health delivery structure of the Punjab Province comprises two ministries: the Primary & Secondary Healthcare Department (PSHD), responsible for primary and secondary level healthcare service delivery, and the Specialized Healthcare and Medical Education Department, which superintends the tertiary healthcare system. PSHD constitutes 33 district headquarter hospitals (DHQs) [27] and 133 tehsil headquarter hospitals (THQs) [28]; together, these facilities offer secondary-level care [29]. Primary-level care is furnished by the Rural Health Centers, Basic Health Units, and dispensaries [29]. Currently, hospital pharmacists are only employed at the secondary care level. However, the medicine procurement process at each District Health Authority (DHA) level occurs for the district’s primary and secondary healthcare facilities and is facilitated by hospital pharmacists. This study was interested in antibiotic procurement at secondary health facilities, for which hospital pharmacists are directly responsible.

### Study design and research strategy

This study utilized an exploratory case study qualitative research methodology [30, 31] to garner hospital pharmacists’ perspectives on antibiotic procurement practices. This type of methodology allows the study of contemporary phenomena in their real-world context and enables the researcher to deeply understand them. Therefore, this research strategy provided deeper insights regarding the case ‘antibiotic procurement process’ within the context of the ‘secondary healthcare sector’ with the single embedded unit of analysis, the ‘hospital pharmacist,’ who is the most appropriate candidate to glean tacit information regarding the subject matter.

### Interview guide

A semi-structured interview guide was used to gather hospital pharmacist’s perspective regarding contemporary antibiotic procurement practices within the secondary healthcare system. An initial interview guide comprising six parts, excluding the demographic section, was formulated. The guide contained open-ended questions that enabled the participants to express their accounts and, at the same time, limit them within the context of our study objectives. An academic expert and a senior pharmacist assessed the initial guide for reliability and content validity. It was then pilot-tested with three pharmacists: two from secondary healthcare facilities and one from the procurement cell of a DHA in Punjab. This process aimed to refine the research questions by gaining insights into the procurement process. The guide underwent important changes after pilot testing, including rephrasing a few questions and the inclusion of new questions as well. Qualitative research is an iterative process involving simultaneous data collection and analysis [32–34]. The updated guide again accommodated a few pertinent questions after ten interviews, which gave a new dimension to the study within the bounds of our ‘case’ and led to the addition of another primary research question. The inclusion of a new objective was considered significant. Following comprehensive discussion within the analytic team (SA, MB, and ZS), consensus was developed to incorporate the new objective for a deeper understanding of the ‘case’ under examination. The interview guide is provided in Additional File 2.

### Development of research question

Initially, the study commenced to understand the antibiotic procurement procedure and to explore how the clinical input of a pharmacist or designated clinical pharmacist can rationalize procurement decision-making. As the interviews progressed, it was realized that participants were more tended to register their concerns about access to antibiotics through the currently implemented procurement system. Therefore, a substantial shift in the dimensions of the study was made after a thorough discussion within the research team.

### Sampling strategy and participant recruitment

The study initially employed purposive sampling until the lead researcher’s network was fully utilized. Subsequently, snowball sampling was implemented, with participants asked to nominate potential participants. Potential participants were telephonically and verbally informed of the study objectives and requested participation. Upon agreement, a formal interview call was scheduled, prioritizing participants’ availability. All participants served as hospital pharmacists at the time of the interview and were posted at different secondary care health facilities situated in different tehsils in the province of Punjab (Table 1). Fifty respondents (33 males and 17 females) were invited to participate in the study during the course of the data collection phase. However, we noticed extreme hesitancy among the respondents regarding participation in the research activity, particularly males; 56% (nonparticipants, 14/25) of them either refused or were unable to fulfil the commitment after agreeing to participate at first due to unknown reasons. Despite this, we could note the accounts of 24 participants in the study, excluding those interviewed during pilot testing. One interview was discarded due to an audio recording issue. The analytical team unanimously decided on data collection termination through data iteration. When it was recognized that within the context of our study ‘case,’ themes have adequately developed and contributed to the comprehensive understanding of our research questions [35, 36]. Data saturation was reached after the 22nd interview; however, further interviews were conducted to confirm theme saturation, which resulted in redundant information. Therefore, data collection was concluded at the 24th interview.

**Table 1:** Participants demographics

Participant ID ^a^	Age	Gender	Hospital Level ^b^	Experience in Public Sector	Qualification	Experience in Procurement
RPh-01	32	Male	THQ	Three Years	M. Phil Pharmaceutics	ThreeYears
RPh-02	32	Male	DHQ	> Five Years	M. Phil in Pharmacology	Four Years
RPh-03	34	Male	DHQ	Six Years	Masters	Six Years
RPh-04	31	Male	THQ	> Five Years	Pharm-D	Three Years
RPh-05	32	Male	THQ	Six Years	Pharm-D	Two to Three Years
RPh-06	34	Male	THQ	Six Years	M. Phil Pharmaceutics	Six Years
RPh-07	31	Male	THQ	Five Years	M. Phil	Four Years
RPh-08	31	Male	DHQ	Six Years	Pharm-D	Five Years
RPh-09	35	Female	THQ	Six Years	M. Phil Pharmacology	Two Years
RPh-10	31	Female	DHQ	FiveYears	M. Phil Pharmacy Practice	Three Years
RPh-11	30	Male	THQ	Seven Years	M. Phil Pharmaceutics	Four Years
RPh-12	38	Male	THQ	Eight Years	MPH	Seven Years
RPh-13	34	Male	THQ	Eight Years	M. Phil Pharmacy Practice	Four Years
RPh-14	28	Male	DHQ	Five Years	Pharm-D	Three Years
RPh-15	34	Male	THQ	Five Years	Pharm-D	Five Years
RPh-16	32	Male	THQ	Four and a half Year	M. Phil Pharmaceutical Chemistry	Three Years
RPh-17	26	Female	DHQ	Two Years	M. Phil Pharmacology	One Year
RPh-18	29	Male	THQ	Six Years	Pharm-D	Five Years
RPh-19	25	Female	DHQ	Two Years	M. Phil Pharmacology	One Year
RPh-20	27	Male	THQ	Four Years	Pharm-D	Two Years
RPh-21	36	Female	THQ	Five Years	M. Phil Pharmaceutics	Three Years
RPh-22	31	Male	DHQ	Five Years	M. Phil	Five Years
RPh-23	33	Male	THQ	Six Years	Pharm-D	Six Years
RPh-24	36	Male	DHQ	Eight Years	Pharm-D	Seven Years

^a^ RPh – Registered Pharmacist, ^b^ Hospital Level – DHQ (District Headquarter Hospital), THQ (Tehsil Headquarter Hospital)

### In-depth interviews

Data were collected via in-depth interviews, predominantly conducted telephonically and audio-recorded, except for one face-to-face interview held in the participant’s office. During a pre-scheduled formal interview call, the participants were again communicated regarding the study objectives, permission to record the interview was sought, and the recording’s purpose was clearly explained for record purposes. The lead researcher (SA, male), holding a Master’s in Philosophy and is trained in qualitative research, conducted all interviews. Field notes were made after each interview. Each participant was interviewed once, lasting between 20 and 60 min, and repeat interviews were avoided. All interviews were conducted in Urdu between October 2023 and March 2024. The audio-recorded interviews were transcribed verbatim and translated into English by NA and JJ. The transcriptions were then proofread and checked for coherence by MSH.

### Data analysis

The data familiarization process started concurrently during the data collection phase by listening to the audio-recorded interviews in full and referring to the field notes. Initially, data were coded and grouped into similar categories that later formed sub-themes, and subsequently, similar subthemes were placed under a single theme. Using the thematic analysis [37], data were initially coded deductively and then inductively for unexpected insights. Two researchers, SA and MB, independently coded the first few transcripts, and an agreed-upon codebook was then finalized after a thorough discussion under the convenorship of ZS, an established researcher who supervised the project. The codebook was then applied to all transcripts, and the analysis was performed using MAXQDA 2024 software. The preliminary findings (draft) were shared with three participants as a member check to invigorate the analysis and check the appropriateness for practice and implementation.

### Ethical consideration

This study was approved by the Human Ethical Committee of the Faculty of Pharmacy, Department of Pharmacy Practice, Bahauddin Zikariya University, Multan (BZU-FOPDPP-2453). All interviewees were assured data confidentiality, and the results were reported anonymously by assigning distinct IDs to each participant. Verbal consent was obtained prior to the interview, and participants could refuse to answer questions or withdraw from the study at any time without explanation.

## Results

**Fig. 1:**
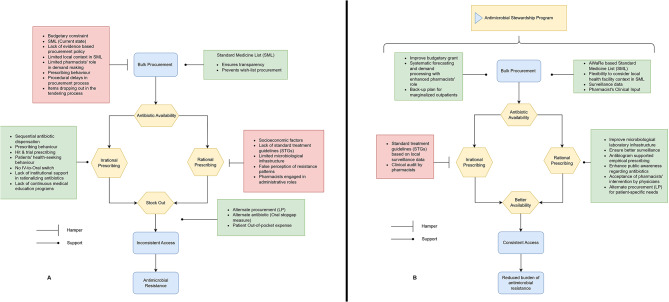
A – Limitations in the current procurement process. B – Recommendations to implement antibiotic stewardship through the procurement process

After careful analysis of the data, eight themes emerged. Five of these are presented in the main manuscript. These include (1) The State of Antibiotic Utilization in Hospitals, (2) Availability Drives Prescribing, Prescribing Drives Availability, (3) The Procurement Process, (4) Rationality in Procurement, and (5) Clinical Pharmacist – A Potential Antibiotic Steward. The examplar quotations for each theme and the remaining three themes are provided in Additional Results, file 1. Table 2 summarizes the findings. Figure 1 (A and B) depicts limitations in the current procurement process and recommendations to implement antibiotic stewardship through the procurement process, respectively. Results are reported according to the COREQ Checklist (Additional file 3).

**Table 2:** Summary findings

Themes	Sub-themes/Sub subthemes	Summary Findings
Pharmacist – The Custodian of Antibiotic Arsenal ^b^	• Understanding of the Concept	• Pharmacists have portrayed limited understanding and awareness of the antimicrobial stewardship program (ASP) and WHO AWaRe classification system. • Notably, only one participant could elaborate on the antibiogram concept.
The State of Antibiotic Utilization in Hospitals	• A System without Antibiotic Policy • Empirical Prescribing – The Default Mode • Broad-Spectrum Antibiotic Use – A Common Practice	• There is a growing trend among prescribers to prescribe broad-spectrum antibiotics empirically without susceptibility testing. • The irrational practice emanates from hospitals’ lack of proper antibiotic use policies.
Availability Drives Prescribing, Prescribing Drives Availability		• Prescribers in public-sector hospitals are encouraged to prescribe antibiotics from the available stock, irrespective of the patient’s specific clinical needs. • The available stock is eminently overprescribed and depletes shortly after it becomes available, thereby limiting the prescribing choice.
The Procurement Process	• Types of Procurement • Budgetary Distribution • Pharmacist’s Role in Procurement • Demand Generation ^a^ • The SML Phenomenon • Pharmacists with 30% Autonomy ^a^ • Lack of Inclusivity in SML ^a^	• There are two broad types of procurement methods: bulk and local purchase (LP) procurement, with budgetary distributions of 75% and 25%, respectively. • Bulk procurement proceeds under the Punjab Procurement Rules (PPR), 2014, using a Standard Medicine List (SML) that specifies medicines and their indicative quantities. • The SML is considered a limitation in catering to hospitals’ medicinal demands, curtails pharmacists’ prerogative in setting demand, and lacks inclusiveness.
Rationality in Procurement	• Antibiotic Resistance Neglect: An Insouciant Procurement Process • “Quantity, instead of Quality” • Access Throughout the Year	• The procurement process does not consider microorganisms’ current resistance patterns or antibiotics’ local susceptibility profile. • The procurement process primarily focuses on ensuring the availability of antibiotics in quantities that could serve the needs of the maximum number of patients receiving treatment within public-sector hospitals. • The procurement process cannot provide consistent access to antibiotics throughout the year, especially in outpatient department (OPD) patients.
Clinical Pharmacist – A Potential Antibiotic Steward		• The pharmacist’s clinical input does not influence procurement decision-making. • Many participants believed that clinical pharmacists can help rationalize the procurement process if involved effectively.
Challenges Besetting the Procurement Process ^b^	• Budgetary Constraints • Irrational Use of Alternative Antibiotics ^a^ • Inability to Procure - The SML Factor • Procedural Delays Affecting Supply Chain • Hospital Pharmacist - An Administrative Role • Irrational Prescribing Affecting Access to Antibiotics • Antibiotics - Drugs of Meagre Importance • Unavailability of Susceptibility Testing • Perception Rather Misconception about the Resistance Pattern	• The most pervasive challenge facing health facilities across primary and secondary healthcare department (PSHD) is budgetary constraint, which has a deleterious impact on access to antibiotics and culminates in the irrational use of alternative antibiotics, thereby augmenting antibiotic resistance. • The limitations presented by the SML and procedural bottlenecks of the procurement process hinder access to antibiotics. • Overuse of antibiotics and empirical prescribing without susceptibility testing are other major impediments. • Pharmacists are overburdened with administrative tasks; hence, they offer minimal patient-centered care. • Policymakers hold a meager understanding regarding the clinical and economic ramifications of antibiotic resistance associated with the misuse of antibiotics.
Recommendations for the Future ^b^	• Nurturing the Clinical Role of Pharmacist • Acceptance in the System • Clinical Pharmacists: Playing a Part in Procurement • “Real-time (Resistance) Data” • Strengthening Clinical Pharmacists with Data • Infrastructural and Budgetary Requirements • Implementation of Antibiotic Stewardship Program • Antibiotic Stewardship – The Vertical Program ^a^ • Clinical Audits across the Facilities • Stewardship Through Procurement • Continuous Medical Education • Adhering to WHO’s AWaRe Classification • Autonomous Procurement	• There is a need to implement an ASP as a vertical program to optimize antibiotic use. • Antibiotic procurement should be conducted under the umbrella of ASP utilizing a data-driven approach, where susceptibility patterns and local epidemiology must be considered significantly. • Strengthening the clinical pharmacist’s role through specialized training and fostering acceptance within the system should be the foremost goal. • Adequate budgetary support for microbiological infrastructure would equip clinical pharmacists with the necessary resistance data to guide antibiotic procurement at the hospital and secretariat levels. • Integrating the WHO AWaRe system with SML would facilitate rational procurement and subsequent antibiotic consumption within hospitals.

^a^ Sub sub-theme ^b^ Detailed results along with examplar quotations are provided in Additional results file 1

### The state of antibiotic utilization in hospitals

#### A system without an antibiotic policy

Antibiotic utilization in hospital facilities suffers from irrational treatment. This is due to the lack of proper antibiotic use policies, giving rise to erroneous prescribing trends divergent from standard treatment guidelines, subsequently posing severe ramifications for the long-term effectiveness of antibiotics.

#### Empirical prescribing – the default mode

It is important to note that the default prescribing practice within the hospital follows empirical or hit-and-trial methods rather than being based on microbial susceptibility testing. Moreover, the lack of a robust data-driven system further complicates the prudent utilization of antibiotics, consequently limiting prescribers to opt for empirical prescriptions.

#### Broad-spectrum antibiotic use – a common practice

The participants highlighted an alarming situation where non-judicious use of Reserve antibiotics from the WHO AWaRe category is on the rise, ignoring their importance as last resort drugs against multi-drug resistant organisms merely to avoid patient complaints. Prescribers also succumb to patients’ perception that injectables are the only effective form of treatment instead of utilizing their clinical acumen and often misemploy broad-spectrum antibiotics, particularly ceftriaxone. Of note, self-medication perpetuated due to socio-economic factors also presents a challenge in rationalizing antibiotics.

Contrary to the popular opinion of antibiotic misuse within secondary healthcare settings, one participant highlighted that prescribers at their facility advocate for rational antibiotic use and express their commitment to ASP. This is encouraging, but such practices are limited across facilities.

### Availability drives prescribing, prescribing drives availability

Public hospitals provide medical services free of cost (or follow subsidized rates for a few services). The provision of medicines to the general public is the hospital’s responsibility, as the availability and provision of medicines within hospitals align with political agendas. Thus, directions are to prescribe medicines from the available hospital inventory and refrain from patients’ out-of-pocket medicine expenditure, irrespective of whether the available antibiotic serves clinical needs. Subsequently, the more prescribed ones are considered during the demand generation. This policy inadvertently promotes irrational use and creates a cycle in which certain antibiotics receive undue preferences in both prescribing and procurement. However, participants mentioned that the alternate procurement method, Local Purchase (LP), is opted to ensure the availability of prescribed medicines or oftentimes oral antibiotics may be used as a stopgap measure, which might not always be clinically appropriate. Moreover, in some scenarios, the patient has to bear the financial burden of purchasing their medicines, which portrays underlying supply chain issues.

On the other hand, the participant explained that there is irrational exploitation of available antibiotics until the stock depletes within a few months, highlighting unguided and non-judicious antibiotic prescribing eventually aggravates AMR.

### The procurement process

**Fig. 2:**
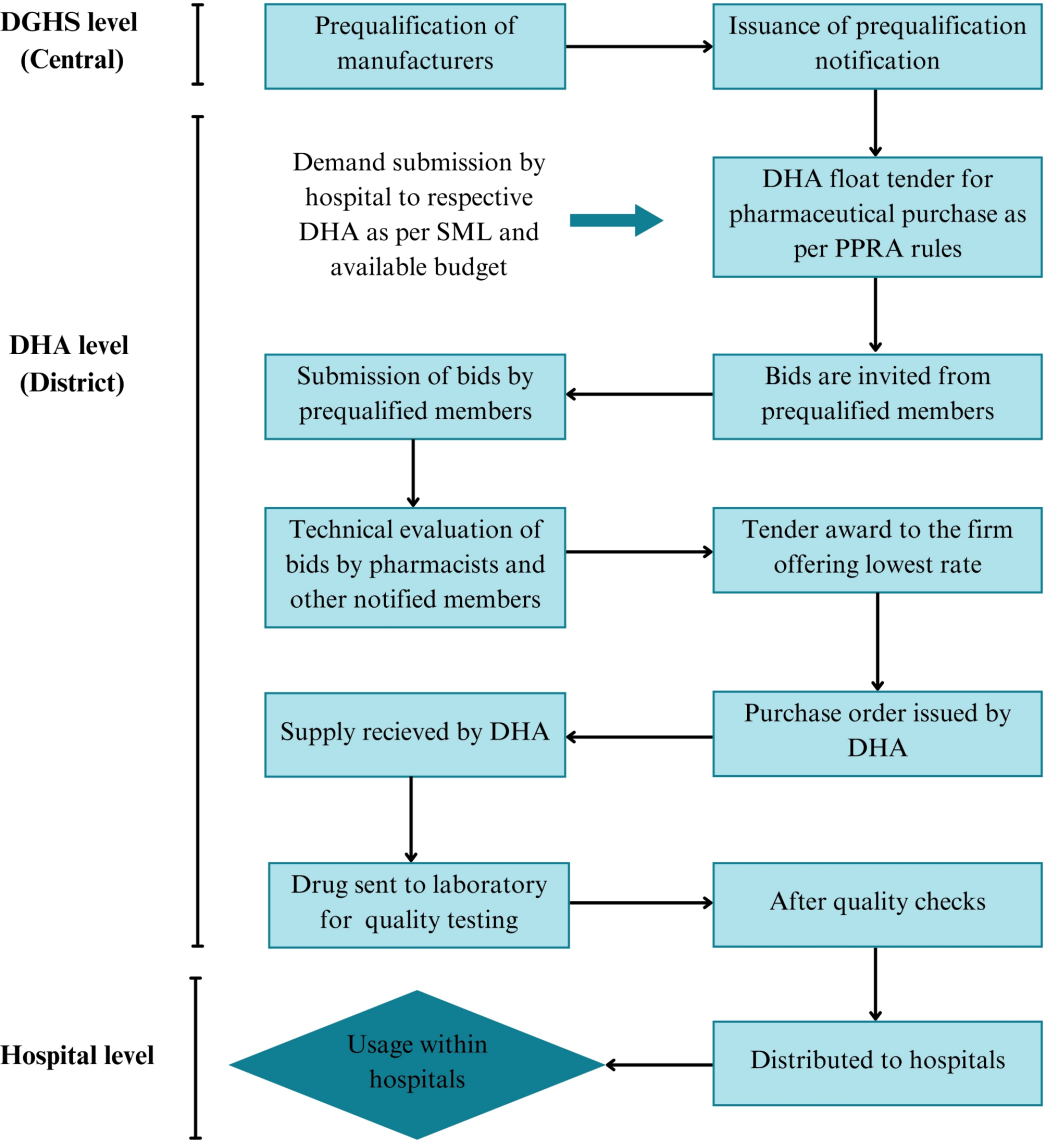
Flow Chart Diagram for Bulk Purchase of Medicines

**Fig. 3:**
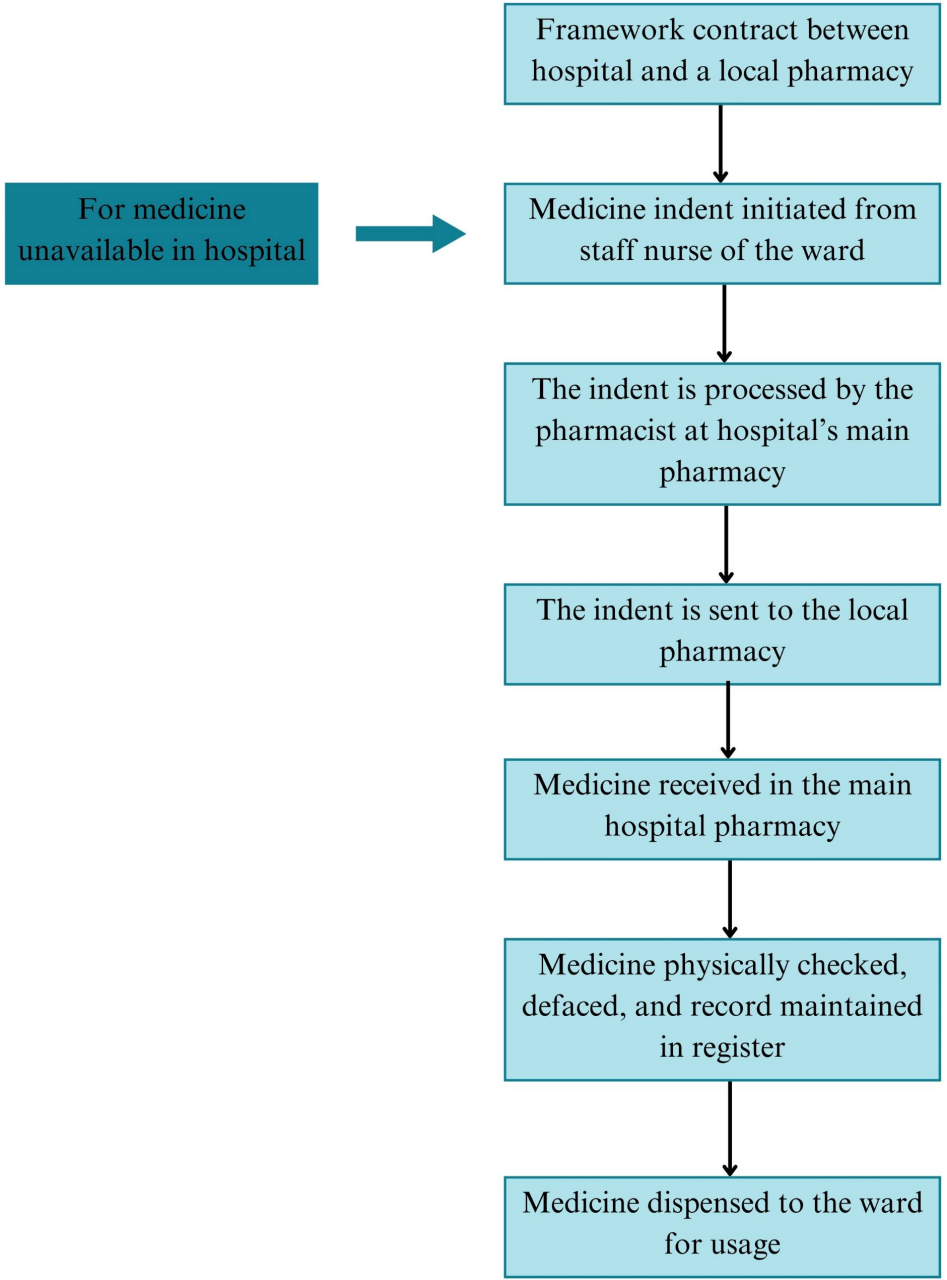
Flow Chart Diagram for Local Purchase of Medicines

Medicine procurement takes place in accordance with the Punjab Procurement Rules (PPR) 2014 set forth by the Punjab Procurement Regulatory Authority (PPRA) as mandated under the PPRA Act, 2009 [38]. Table 3 provides different attributes of the procurement process. Figure 2 demonstrates the bulk procurement process in a flow diagram. Additionally, Fig. 3 depicts the procedure for local purchase (LP) taken place at the hospital level.

**Table 3:** Procurement attributes in a secondary healthcare system

Types of Procurement	• Bulk Procurement	• Local Purchase (LP)
**Budgetary Distribution**	• 75%	• 15% - Day-to-day Purchase • 10% - Emergency and Disaster Management
**Pharmacists’ Role in Procurement**	• Demand Generation at the hospital level • Technical member of the district purchase committee	• Demand processing • Receiving medicine from a local vendor and physical verification of the stock
**Factors Involved in Demand Generation**	• Previous year’s consumption • Prescribing trend • Allocated budget • Standard Medicine List (SML)	• Items unavailable in bulk procurement

#### The SML phenomenon

The Standard Medicine List, commonly referred to as ‘SML,’ is notified by the procurement cell of the DGHS. SML is a list of medicines along with their indicative quantities, in which health facilities of both primary and secondary nature are categorized according to their bed strength (Additional file 4). As per clause 5 of the SML notification, the PSHD has made it mandatory to follow the list and quantities therein for the bulk purchase of medicine as a policy decision [39].

The participants predominantly disrelished SML. They thought that SML bound us within prescribed limits, allowing alteration of up to ± 30% in demand and asserting rigid adherence, rendering it arduous to cater to the medicinal requirements of the hospital. One of the participants argued that substantial adjustments in the hospital’s actual consumption had to be made to meet the SML criteria, questioning its formulations’ effectiveness for every facility. The participants extended their argument by mentioning that facility-to-facility variability in demand is evident, stating that SML disregards this factor and manifests less usefulness overall.

On the contrary, few participants supported SML and held a balanced opinion on its constitution. The participant described that the list appreciably enumerated emergency and indoor medicinal items in the first place, which reflected the government’s priority to provide for these departments. Moreover, SML includes a wide variety of antibiotics but still requires amelioration. The participant further admitted that SML was deficient in providing full coverage due to the high resistance pattern of a few antibiotics. However, this lack has been defended by the participant, who elaborates that the government expects to cater to such patient-specific needs through the budget of LP.

### Rationality in procurement

#### Antimicrobial resistance neglect: an insouciant procurement process

Almost all participants unanimously confirmed that the procurement process was inconsiderate towards the resistance pattern of either the province or presented in the hospital. Furthermore, no such data regarding the resistance pattern is available, which could assist in rationally selecting antibiotics in the procurement process. Such an insouciance in procurement could produce grave concerns regarding AMR.

The participant asserted that AMR is never a concern in procurement. However, a participant presented an absurd justification, claiming they determined the resistance from the prescribing pattern observed within the facility.

Contrastingly, only one participant argued that resistance status was considered in the procurement process, particularly during pre-qualification.

#### Access throughout the year

Most participants rejected the notion that access to antibiotics is ensured year-round in government settings. Furthermore, sequential antibiotic consumption maximizes stock levels but limits prescribing options and downplays resistance considerations, restraining effective patient care and raising grave concerns about AMR.

The participant explained that access to medicine is distinct for outpatient department (OPD) patients versus in-patient/admitted patients with in-patient receiving medicine/antibiotics through alternative procurement methods. However, outpatients are at a disadvantage and face disparities in antibiotic availability, pointing towards underlying supply chain issues, poor resource allocation (for outpatients), and inequitable healthcare provision. Furthermore, a participant hinted that the rational use of medicines or antibiotics is still debatable, even in admitted patients. Access to medicine cannot be comprehended as a mere logistical element of medicine availability; rather, rational use of medication is equally important [40].

### Clinical pharmacist – a potential antibiotic Steward

In the present regime, the clinical pharmacist himself or the clinical acuity of any pharmacist cannot affect the procurement of antibiotics, most of the pharmacists asserted. The predominant reason stated by the participants was the lack of data pertaining to the resistance pattern of the province and, subsequently, the hospital facilities. Moreover, the current procurement process is not inclusive enough to consider the clinical role of pharmacists due to budgetary constraints, pharmacists’ engagement in administrative roles, non-availability of clinical pharmacists, and limited physician acceptance of pharmacist intervention.

However, all participants unanimously acknowledged that involving a clinical pharmacist in the procurement process will significantly improve antibiotic rationalization and reduce the cost incurred on antibiotics.

## Discussion

Our findings suggest inconsistent access to antibiotics accompanied by an arbitrary selection of antibiotics without considering the susceptibility pattern of the microbes, and the same is also disregarded during the procurement process. Moreover, pharmacists’ clinical expertise concerning antibiotic procurement is underutilized due to insufficient susceptibility data and challenges in the form of budget and physician acceptance.

Access to antibiotics is closely connected to the utilization pattern and budgetary grants within health facilities [41]. An erratic and irrational prescribing by physicians disparaging supply chain issues and budgetary limitations eventually results in early stockouts of the antibiotics being purchased through bulk procurement, a predominant method used to cater to the hospital’s medication needs, as noted in this study. Pharmacists can play an integral role in managing stockouts, performing the dual role of medication management at the patient-care level and as an inventory manager [42]. However, our study results suggest a contrasting picture where a pharmacist’s clinical acumen is rarely employed and thus offers negligible utility in patient-centered care.

The process of procuring medicinal items within the healthcare department follows a semi-centralized approach, and pooled procurement is based on SML as a policy decision. Although the National Essential Medicine List (EML) 2023, notified by the Government of Pakistan [43], and generally, these types of formularies or lists ensure transparency and prohibit the inclusion of wish-list items for procurement and are considered elements of good procurement [44, 45]. However, the indicative quantities of antibiotics enumerated on SML do not represent the actual needs of health facilities. Instead, the participant accentuated that the actual demand for the facility had been adjusted for up to 50% to comply with SML-notified quantities. The present policy negatively affects access to antibiotics [11] and undermines the pharmacist’s role as a procurer (medicine quantification, forecasting, and demand management), in contrast to empirical evidence that a pharmacist’s clinical and managerial acumen regarding procurement is highly appreciated [42, 46–48].

Hospital pharmacists manage bulk procurement inadequacies by opting for an alternative procurement method, LP, to arrange the medication from a local vendor. However, facility-to-facility budgetary hardships also hinder such an arrangement in the long run. This channel can potentially expose public health facilities to substandard medicine supplies [49, 50]. Hence, the quality of the antibacterial agents supplied through this channel is a topic that requires further exploration.

It was observed that larger facilities such as DHQs tend to have more budgetary space than THQs and can afford alternate procurement for in-patients and emergency departments; however, outpatients are at a disadvantage in both types of facilities once the bulk supply of antibiotics is depleted. Contrarily, the participant also highlighted that budgetary shortages compel physicians to choose inappropriate alternatives available in the hospital. A previous Pakistani study [22] indicated poor procurement procedures as the cause of medicine shortages. The study recommended extra-budgetary allocation and strengthening pharmacists’ role in procurement to ensure optimal access and avoid hospital medicine shortages. A study from Ethiopia [51] similarly found that budgetary shortages affected the availability of medicinal products. Overall, this study also noticed that budget directly influences access, quantity procured, prescribing, and clinical intervention by pharmacists, and vice versa.

This study also corroborates previous findings that LMICs lack microbiological laboratory infrastructure and data surveillance capacity [52, 53]. Pakistan’s NAP [20] also acknowledges that such weaknesses exist in the country’s healthcare setup. The participants asserted the need to develop a microbiological laboratory infrastructure to facilitate definitive antibiotic therapy and to restrain arbitrary empirical treatment. Antimicrobial susceptibility testing systems will guide local antibiogram development, support appropriate initial empirical prescribing, establish treatment guidelines, and inform emerging resistant trends [54–56].

Unfortunately, the study participants were unfamiliar with the concept of antibiograms, except for one participant, and the determinant of this finding could be explored further in light of the pharmacy curriculum. Therefore, the term “resistance data” has been utilized alternatively while interviewing participants. In our study, participants unanimously suggested that antimicrobial resistance is not considered while procuring antibiotics because of the absence of resistance data surveillance across the health facilities. They indicated that a data-driven approach would be highly suited for antibiotic procurement. Published literature suggests that pooling resistance data from different health institutions across vast geographical regions, such as provinces, and synthesizing such data to form an accumulative antibiogram could serve as an essential guiding tool for appropriate empirical therapy and current resistance trends [31, 57–59]. Building on the literature, antibiograms or susceptibility data collected from facilities across the province can help identify appropriate antibiotics for subsequent inclusion in the procurement list [54, 60]. Such an antibiotic list based on accurate representative AMR data would provide substantial evidence for procuring antibiotics [61], unlike SML [26], which does not consider current resistance patterns in selecting antibiotics and thus appears to be divergent from the updated evidence base.

Our study found a lack of standard treatment guidelines (STGs) for antibiotic prescribing within the province’s secondary healthcare system, contrary to the claim made in Pakistan’s NAP against AMR regarding the development of STGs within the country [20]. The foremost stewardship intervention is to formulate an antibiotic prescribing policy that informs the contextualized selection of antibacterial agents based on the local susceptibility profile, which will ascertain an appropriate empirical prescribing [15, 52]. Moreover, The Medical Microbiology and Infectious Diseases Society of Pakistan [62] and the WHO AWaRe framework offer practical guidance regarding empiric prescribing upon which local guidelines can be based [63], or the WHO AWaRe Antibiotic Book can be adopted as a comprehensive empiric prescription-guiding tool that covers 34 common infections present in primary healthcare and hospital settings [64, 65]. However, neither this study noticed any consideration to opt for any prescription guiding tool within the public health setups, nor did Atif et al. [29] and Butt et al. [66] Furthermore, because of the absence of antibiotic guidelines and poor diagnostic abilities of the public health sector, as outlined in NAP Pakistan [20], 100% of the antibiotic prescriptions were constructed on an empirical basis, as noted by Mustafa et al. at secondary care public hospitals [67], similar to our results. As an outcome, irrational practices prevail, leading to the use of broad-spectrum antibiotics, particularly of the Watch and Reserve category from WHO’s AWaRe Classification, as observed by this study. Clearly outlined bespoke antibiotic guidelines will eventually facilitate judicious prescribing, appropriate demand forecasting and rationalized procurement [16].

Our results suggest that the procurement process does not engage clinical pharmacists or utilize their clinical understanding in the appropriate selection of drugs for procurement, forecasting, and demand formation for various reasons discussed in the above sections. The most notable one is that neither the clinical pharmacist is supplemented with sufficient susceptibility data to present an argument regarding the purchase of antibiotics, nor does the budget provide space for the pharmacist to intervene and recommend alterations clinically. An extensive literature base is available regarding the effectiveness of antimicrobial optimization, therapeutic guidelines, formulary development, and proper implementation of ASP by clinical pharmacists [68, 69]. However, the lack of STGs and microbiological laboratory infrastructure was noted to be the major impediment to the propagation of their role as antibiotic stewards [70]. Fortunately, many facilities, with few exceptions, under the administrative control of PSHD have a designated Clinical and Pharmacovigilance officer (CPPO) responsible for medicine optimization and reporting drug-drug interactions. There is a need to expand their role in implementing ASP to ensure rational utilization of antibiotics within their facility through prospective audit and feedback [71] while guaranteeing timely antibiotic access through good procurement practices will ultimately slow AMR. However, to take adequate advantage of this enabler situation, their role must be recognized, and acceptance must be generated within the system.

A well-designed procurement module is essential to ensure consistent access to antibiotics. It should be embedded as a critical component of the ASP, which is crucial for the timely availability of antibiotics to improve patient outcomes [11, 17, 18]. Consistent with the literature, participants rightly emphasized the need to implement an ASP and conduct procurement of antibiotics within this framework. Moreover, another participant recommended establishing the ASP as a separate vertical program with the purview of procuring medicine to tackle AMR effectively. This approach can be further debated, as the existing literature illustrates the positive effects of vertical programs for focused antimicrobial stewardship activities [72–74]. The WHO’s AWaRe framework is also an antimicrobial stewardship tool that can equally serve to monitor and evaluate access to antibiotics, as the recent United Nations General Assembly (UNGA) high-level meeting on AMR recommends that by 2030, 70% of antibiotic consumption at the global level should be of Access group antibiotics [75–77]. Participant eloquently suggested that procurement (SML) must be devised in such a way that compliance may possibly be generated. Even though the National EML 2023 classifies antibiotics as per the WHO AWaRe list, unlike SML [43]. Unfortunately, NAP Pakistan [20] is also silent on standardizing antibiotic procurement within public health entities and integrating the WHO AWaRe system within the antibiotic procurement process.

### Strength and limitations

The major methodological limitation is that the case-study qualitative methodology is context-specific. Therefore, results lack generalizability and are limited to the ‘case’ and ‘context’ under discussion. The study counts this as a limitation that only the perspectives of hospital pharmacists were noted. This study primarily addressed antibiotic access issues through the current procurement system. So, in this regard, the prescriber is another important stakeholder. Considering the perspectives of this key informant would enable us to understand antibiotic access more nuancedly in PSHD. On the other hand, this study established substantial grounds related to procurement procedures followed in the public health sector, particularly the domain of hospital pharmacists, and gathered significant empirical evidence to inform policy and design measures to promote antimicrobial stewardship through procurement.

Pharmacists exhibited hesitancy in participating, possibly because of the perceived displeasure of authorities and disobedience of service rules; however, the interviewed participants were vocal and candid about their concerns and articulated their perspectives fairly well, contributing to an in-depth understanding of the research questions.

Incorporating quantitative procurement data could significantly enhance the study’s depiction of antibiotic procurement. The research team is developing a mixed-method project to triangulate this qualitative data with quantitative figures, providing a more nuanced explanation of antibiotic procurement and related aspects.

### Practical implications

The study offers sufficient evidence for stakeholders to reassess the current SML policy on antibiotic procurement and suggest its alignment with the WHO AWaRe classification and the National EML 2023. Current practices are unable to ensure consistent access to antibiotics. Moreover, disregarding the provincial and hospital antibiotic susceptibility patterns during procurement, coupled with a lack of antibiotic guidelines results in irrational procurement and inappropriate (misuse and overuse) utilization of antibiotics within the facilities. Data from local microbiology or pathology laboratories or the WHO Global Antimicrobial Resistance and Use Surveillance System (GLASS) can be used for susceptibility profiles [31]. However, care must be taken when synthesizing results from such surveillance data due to the possible risk of bias, poor quality, and lack of representativeness [78–80]. This study advocates the efficient utilization of clinical pharmacists within the procurement domain for appropriate drug selection and rational demand forecasting based on susceptibility patterns and disease epidemiology. For this purpose, the pharmacist’s clinical role must be signified within the secretariat, DHAs, and, importantly, hospital settings.

## Conclusion

Antibiotic procurement is the mainstay of implementing an ASP. Consistent access to antibiotics is subject to good procurement practices and, subsequently, their responsible utilization within hospital settings, according to clinical guidelines. The study identified the negligible clinical role of pharmacists in rationalizing the procurement process, along with other associated challenges. Pragmatic recommendations to overpower the challenges of access to antibiotics, procurement policy, and the development of the pharmacists’ clinical role have been provided to promote stewardship program through rationalized antibiotic procurement.

## Electronic supplementary material

Below is the link to the electronic supplementary material.

Supplementary Material 1

Supplementary Material 2

Supplementary Material 3

Supplementary Material 4

## Data Availability

The datasets generated during and/or analyzed during the current study are available from the corresponding author on reasonable request.

## Abbreviations

ASP: Antimicrobial stewardship program
LMICs: Low- and middle-income countries
AMR: Antimicrobial resistance
SML: Standard Medicine List
SDG: Sustainable Development Goal
WHO: World Health Organization
GAP: Global Action Plan
NAP: National Action Plan
PSHD: Primary & Secondary Healthcare Department
DHQs: District headquarter hospitals
THQs: Tehsil headquarter hospitals
DHA: District Health Authority
LP: Local Purchase
PPR: Punjab Procurement Rules
PPRA: Punjab Procurement Regulatory Authority
DGHS: Directorate General Health Services
RFP: Request for Proposal
CEO: Chief Executive Officer
OPD: Outpatient department
IPD: Inpatient department
CPPO: Clinical and Pharmacovigilance officer
STGs: Standard treatment guidelines
UNGA: United Nations General Assembly
