# Supplementary material for: The antibiotic procurement saga: a long-neglected stewardship target to combat antimicrobial resistance in Pakistan
Source: Antimicrob Resist Infect Control. 2025 Feb 7;14:7. doi: 10.1186/s13756-025-01521-w (PMC11806573; doi:10.1186/s13756-025-01521-w)
Supplement: Supplementary file 1 — Supplementary Material 1 [file 13756_2025_1521_MOESM1_ESM.docx]

**The Antibiotic Procurement Saga: A Long-Neglected Stewardship Target to Combat Antimicrobial Resistance in Pakistan**

**Supplementary Results**

- 1. **The State of Antibiotic Utilization in Hospitals**
     1. ***A System without an Antibiotic Policy***

*“Actually, the matter is in our secondary care hospitals, where there is limited sort of work on the antibiotics policy. Now, it is my personal view that until we don’t have an antibiotic policy, we can’t develop an effective rationale” RPh-12*

*“Our doctors have the prescribing trends that they will not start from the first-line antibiotics but instead prescribe third-generation antibiotics such as moxifloxacin or other advanced antibiotics, which results in the ineffectiveness of other antibiotics.” RPh-21*

- - 1. ***Empirical Prescribing – The Default Mode***

*“The major thing is that whenever an antibiotic is to be prescribed, it should be according to the culture and sensitivity test. But in the current scenario, every therapy is empirical. (---). So, it is just a hit-and-trial method because we do not have any data (patient history). Any antibiotic that responds to the patient is included in the treatment.” RPh-18*

- - 1. ***Broad-Spectrum Antibiotic Use – A Common Practice***

*“We directly jump to life-saving drugs, and now, even linezolid use is also on the rise. Orthopedic and surgery departments use it excessively because they do not want to have any complaints from their patients.” RPh-13*

*“First of all, we have to see whether he requires antibiotics or even ceftriaxone. Nobody considers this; it is just that you have to inject him something so that the patient is satisfied that he has been treated.” RPh-9*

*“Inflation plays a major role in this process. Right? And now, it has become a trend that due to self-medication, they have become resistant to a wide variety of antibiotics. Owing to this, we have to change the generations and move to higher generations or use combinations instead of plain salts.” RPh-5*

*“We have new doctors and pediatricians. They specifically work to achieve antimicrobial stewardship goals and advocate that antimicrobials, such as azithromycin, should be saved for later.” RPh-23*

- 1. **Availability Drives Prescribing, Prescribing Drives Availability**

*“The government says to prescribe those medicines that are available in hospitals. Because every antibiotic is the same for them. (---). So, the available one is prescribed by doctors and is subsequently considered while generating demand.” RPh-11*

*“But I would repeat it that in government hospitals, the trends in medicines are based on the availability of medicines. (---) and not based on patient needs. So, if, for example, we have ciprofloxacin injections available in the hospital, they (doctors) will continue to administer it even if the patient complains of palpitations and arrhythmias. But since only ciprofloxacin is available in the hospital, they will continue it as a broad spectrum (antibiotic).”* *RPh-12*

*“If the items are not available (in the hospital) and the demand is from the department (ward), we shift it to the LP. (---) and is forwarded to the LP vendor, who in turn provides supplies of the same items to the department.” RPh-19*

*“As a first resort, we try to provide the antibiotics through local purchase (LP) if there is a shortage in the bulk. Otherwise, we also prescribe oral antibiotics to compensate for the deficiency of injectables. But if we have no other option, the patient has to bear the expenses.” RPh-18*

*“I purchase almost 5000-7000 units of Cefixime suspension for the peads’ OPD. What the doctors do is they prescribe it to almost every patient, as a trend. They keep prescribing it until in three to four months, we have no supplies left.” RPh-13*

- 1. **The Procurement Process**
     1. ***Types of Procurement***

Medicine procurement takes place in accordance with the Punjab Procurement Rules (PPR) 2014 set forth by the Punjab Procurement Regulatory Authority (PPRA) as mandated under the PPRA Act, 2009 [1]. Directorate General Health Services (DGHS), under the administrative control of the PSHD, initiates the pre-qualification process of the manufacturers as per Rule 16 of PPR 2014’ for items listed under the Standard Medicine List (SML). DGHS notifies the list of prequalified suppliers, which is followed by DHAs across Punjab, initiating a pooled procurement process and inviting a ‘Request for Proposal’ (RFP) from the prequalified manufacturers to submit the bid. The bidding complies with the single-stage two-envelope procedure, in which technical and financial bids are submitted separately in a single sealed envelope. The Chief Executive Officer (CEO) of DHA notifies the technical evaluation committee, encompassing primarily pharmacists, to evaluate the bids on technical grounds as per a pre-defined criterion disseminated under RFP. Technically sound bids proceed further and are subject to the opening of their corresponding financial bids. The manufacturer offering the lowest rate against an item is awarded a contract. **(Figure 1)**

*“This department (DGHS) has been given the mandate or, should I say, delegated the power to pre-qualify the firms every year for bulk purchases. Obviously, they perform the pre-qualification for uniformity and quality and they do it via a ‘request to pre-qualification’ method. This is performed according to rule no. 16 of the PPRA rules (---). Once the pre-qualification is completed, the CEO of that particular district health authority passes a direction to the hospitals under his administrative control for the generation of demand.” RPh-12*

Participants explained that there are two types of purchases made for procuring medicines: bulk purchase, as described above, and local purchase (LP) at the hospital level, as a countermeasure to cater to the unavailability of any medicinal item in the bulk purchase. Wherein a vendor is engaged in a framework contract under Rule 15(1) of the PPR 2014 [1]. The hospital is responsible for advertising newspaper tender requests to invite bids from potential vendors. The contract was awarded to a vendor offering a maximum discount on the medicines supplied. Predominantly, these two procurement methods prevail to cater to the medicinal needs of the hospital. **(Figure 2)**

*“Two types of purchases are carried out: bulk purchase and local purchase. (---). We have a specified local vendor through whom we purchase routinely used medicines that are not provided to us in bulk (purchase).” RPh-01*

*“This (LP) contract is awarded to a specific vendor through a tender under a framework contract for the LP.” RPh-12*

- - 1. ***Budgetary Distribution***

The participant explained the budget distribution allotted to public hospitals in the following quote.

*“We use 75% of it (this budget) for bulk purchasing through tenders, right? And it is done by the DHA. The remaining is 25%, of which 15% is utilized for day-to-day (Local Purchase). (---). The remaining 10% is reserved for emergency purposes.” RPh-04*

- - 1. ***Pharmacist’s Role in Procurement***

At the district level pharmacists serve as technical members of district purchase committees formed exclusively for bulk medicine procurement. Their role encompasses demand generation, technical evaluation of dossiers in the tendering process, and physical verification of supplies. They are also involved in other procurement-related matters; however, most participants claimed they were not part of the financial proceedings.

*“We are members of the Technical Evaluation (Committee).” (RPh-03)*

*“So, I cover almost all parts of the procurement except for financial matters or financial bidding, which is not linked to us, but I oversee all the technical matters.” (RPh-05)*

- - - 1. ***Demand Generation***

The demand-generation process involves multiple intricacies; however, the prevailing factors include the previous year’s consumption, the prescribing trend, the allocated budget, and, most importantly, the SML.

*“One thing is that there is a standard medicine list of hospitals. Secondly, we see the trend of prescribers in our hospital; what are their prescribing habits? .(---). So, the antibiotic in high demand is procured in large quantities.” RPh-24*

- - 1. **The SML Phenomenon**

*“We follow that list exclusively and generate the demand within that list, keeping in mind the previous year's consumption and budgetary requirements. Things such as rational use or actual need in the hospital are rarely considered.” RPh-09*

*“Now, the standard medicines list SML that the government of Punjab has developed, I think it needs a lot of improvement. It has many flaws.” RPh-10*

*“I processed the demand by adjusting almost 50% of the medicines to meet the SML criteria. So, it is not that kind of list designed by considering each hospital of the province or the local population. (---). Therefore, the SML is not very helpful.” RPh-22*

*“It (SML) basically targets emergency and inpatient department (IPD) items. (---). Secondly, they are also trying to cover the maximum number of antibiotics in it. However, there are some gaps as well. (---). We require several antibiotics because of the high resistance of other antibiotics, but they are not on the list. But, on the other hand, when they leave these gaps, they also know that we have a side budget (LP) as well, and it’ll cover the gap on the list.” RPh-19*

- - - 1. ***Pharmacists with 30% Autonomy***

Another participant mentioned that they could only alter their demands by ± 30%. Moreover, they cannot choose antibiotics outside the SML, which could have significant repercussions on patient-specific needs and resistance patterns. The quote shows that SML curtails pharmacists’ prerogative in making independent decisions regarding the hospital’s needs and procurement of antibiotics, which is analogous to questioning their professional judgment.

*“The only alteration that can be made in the SML is to increase or decrease the quantities by up to 30%, based on our hospital's consumption. (---). So, we have to choose the antibiotics according to the SML.” RPh-17*

Furthermore, when procuring any item outside the SML or manipulating demand beyond the confined limits, special requests along with justification must be made when planning the procurement to the DGHS. After due diligence, a committee decides the fate of the request. A reservation was shown by the participants regarding the acceptance of their request.

*“If we want to procure something from our own, we must justify it. However, the ball remains in the (DGHS) pocket whether to allow or disapprove. In the latter case, we must confine ourselves within the bounds of that list while generating demand.” RPh-19*

- - - 1. ***Lack of Inclusivity in SML***

The comment below elaborates that the decision to formulate SML involves little input from related stakeholders; instead, most of the decisions are made based on administrative understanding. Therefore, its formulation represents minimal inclusivity. On the contrary, the SML notification claims that an expert working group involving different stakeholders contributed to its composition.

*“When the procurement is carried out or the standard medicine list is prepared, they (DGHS) are unable to take the input from everyone. Very few people were involved in it.” RPH-08*

- 1. **Rationality in Procurement**
     1. ***Antimicrobial Resistance Neglect: An Insouciant Procurement Process***

*“No, not at all. (---) There is nothing as resistance in the procurement process. They just tell us that this is the standard medicine list; it has the following antibiotics, which must be available in your hospital in these quantities irrespective of whether it is resistant or not, whether it would have an impact on the patient or the opposite, and also how much would it cost us in bulk or whatsoever.” RPh-05*

*“The role of resistance comes in the procurement process indirectly, not directly. (---). It is observed from the prescribing habits that if an antibiotic is* *highly* *prescribed, then it has a high susceptibility rate. If it is prescribing less, it is either resistant, or the prescriber is not taking the results out of it. Therefore, such antibiotics are consumed less and have fewer prescriptions. (---). Basically, we are conducting procurement based on demand.” RPh-06*

*“They do consider this because when they (DGHS) carry out pre-qualification, they would surely have included some of the molecules in the list derived from the resistance trends. (---). However, the scale of the consideration can be debated.” RPh-12*

- - 1. ***“Quantity, instead of Quality”***

The participant highlighted that the quantity of the medicine or antibiotic is focused on more than its rational use or individualized patient requirement during the procurement process. In government settings, administration of some forms of medicine is always necessary to satisfy the patient. Thus, patient coverage and economic considerations are primarily considered when procuring medicine. This approach has a fair share of implications for AMR prevalence and rational utilization of antibiotics.

*“Economical antibiotics will be procured more in quantity, and public sector hospitals will focus largely on quantity. This means, at maximum, how many patients can be covered, how many will visit the hospital, and how many can be made to utilize the antibiotics effectively? Or any other medicine.” RPh-02*

- - 1. ***Access Throughout the Year***

*“No, not at all. I state with a firm conviction that (---) you (pharmacist) issue an antibiotic, wait for its depletion, then issue a second one and wait for it to deplete and then the third one, and then the fourth one. So, ‘choice’ and ‘resistance’ are just out of the question. (---) You can say that ideally, there are only two months in the whole year when the patients have full access to antibiotics, and the prescribers have the choice to choose between multiple antibiotics according to the needs of their patients.” RPh-13*

*“I would say sometimes, when we do not have timely supplies, we face constraints in the OPD. We want the patients to have these antibiotics, but the antibiotics are not available, and the patients cannot afford them. Therefore, this is definitely a parameter that affects patients. However, when we talk about admitted patients, they are largely facilitated. Antibiotics are made available at the maximum through the bulk or LP and administered immediately. You can discuss the medicines being administered, those that should or should not be administered, the dosing, or whatever other things in these patients. But yes, the OPD patients are compromised to some extent because we cannot facilitate them through the LP. They can only be facilitated by bulk medicines.” RPh-19*

- 1. **Clinical Pharmacist – A Potential Antibiotic Steward**

*“The clinical pharmacist is not a part of the procurement yet.” RPh-17*

*“In some places, such as my hospital, there are five vacancies for pharmacists, but I am the only one working as a pharmacist. Seats are still vacant. (---). So, the procurement process is not broad in terms of antimicrobial resistance status.” RPh-05*

*“See, the authorities at higher levels want the pharmacist to play his role, but some of our clinicians do not allow this because they are stuck to their prescribing habits. (---). So, no doubt that there is no flexibility in a system as such till now.” RPh-06*

*“Clinical pharmacists must have sufficient grounds or clinical data to present regarding the more or less purchase of antibiotics. Personally, I do not think that there is any pharmacist in the whole of Punjab who gathers a pool of data that he can present as an argument to tell the resistance status of antibiotics. He did not have data on resistance, feedback from patients, or even the original feedback of the prescriber.” RPh-13*

*“See, SML is implemented throughout Punjab. (---). I don’t think so much input from a clinical pharmacist is considered.” RPh-11*

*“When we give our opinion on such (clinical) matters, we face limitations from our financial resources. The doctors will say, ma'am, if you think the other option is better, why don't you procure it for us? So, here again, budget is a limiting factor.” RPh-21*

*“The process will be improved if we use pharmacists' clinical services in procurement.” RPh-22*

- 1. **Pharmacist – The Custodian of Antibiotic Arsenal**
     1. ***Understanding of the Concept***

Most participants claimed that their university education contributed to their knowledge regarding antibiotics, and currently, they update themselves by using various online platforms. Moreover, interaction with doctors and consulting relevant books were also reported to be a predominant source of information.

*“The basic knowledge of antibiotics is from the pharmacy degree. Apart from that, I see new antibiotics when they are marketed from time to time or when our doctor or consultant prescribes such antibiotics. Moreover, I consult Lexicomp or Medscape apps in case there is an issue with the dose or other.” RPh-07*

The present study elucidated that pharmacists possess a partial understanding of the concept of antimicrobial stewardship program (ASP). In fact, most participants were confused about familiarity with the term ASP compared to their actual understanding of the program. However, only a few participants could explain the term in its true sense.

*“Yes, I do have the idea. Antibiotic stewardship, as the name indicates, (---), I mean the main crux is related to how we can minimize the use of antibiotics and use them judiciously and appropriately. which is its main theme.” RPh-02*

The study noted the participants' sparse awareness of the WHO’s AWaRe classification system. Only a few participants were able to elaborate on this concept.

*“A document was shared with me regarding WHO’s AWaRe classification. I gave it a shallow read. (---), but I do not have an exact idea about the classification.” RPh-19*

However, only one participant was familiar with and could elaborate on the concept of an antibiogram.

*“Antibiogram, yes. It is like a graph where the written notes about the local resistance pattern of antibiotics as well as the resistance of the current microbiological flora related to the disease are provided.” RPh-22*

- 1. **Challenges Besetting the Procurement Process**
     1. ***Budgetary Constraints***

All participants unanimously declared budgetary constraints to be the most pervasive challenge looming across Punjab’s health facilities. Every participant discussed financial hardship relative to the problems one was facing in their institution. The participant expressed in the quote below that a budgetary grant does not adjust for the inflationary rate corresponding to which the lower quantities of antibiotics could be purchased, resulting in limited access to antibiotics. Furthermore, the facility cannot procure a few essential antibiotics again, halting access to antibiotics.

*“Day by day, the cost is increasing while the budget stays the same, so the number of antibiotics, i.e., their quantity, is reducing. (---). In addition, we are unable to procure advanced antibiotics like vancomycin, meropenem, and piperacillin/tazobactam. (---). These are costly antibiotics, and our budget can’t afford them.” RPh-07*

The participant below discussed that the number of registered patients in the outpatient department (OPD) is increasing, as is the number of inpatients. However, budgetary hardships prevent them from procuring broad-spectrum antibiotics and instead limit them to low-cost, narrow-spectrum items that are unable to provide sufficient antibiotic coverage as per patient needs.

*“We have an OPD of 4000 patients daily, and the number of indoor patients and admissions is quite high. (---). For this reason, the first and foremost challenge that we face is the budget and budgetary constraints. We can’t procure the latest items; instead, we try to procure low-cost items in which we often overlook the basic patient needs.” RPh-08*

The participant acknowledged in the quote below that financial constraints affected access to antibiotics.

*“You can say it does not ensure 100% access. We can only provide access to antibiotics for about 70 to 80% of the annual duration. Due to financial constraints, we are unable to take it to 100%.” RPh-22*

- - - 1. ***Irrational Use of Alternative Antibiotics***

The quote below highlights a concerning situation where budgetary limitations impact antibiotic selection. Physicians are compelled to choose less suitable alternatives or sometimes resistant antibiotics, disregarding the optimal patient care standards. The situation presents an ethical dilemma where budgetary imperatives are prioritized, compromising appropriate antibiotic selection and transgressing optimal patient care.

*“Yes, it's true that due to budget limits, inappropriate alternatives are chosen. Similarly, sometimes resistant antibiotics are also used because doctors say they should avoid vancomycin, meropenem, linezolid, and other related salts due to budgetary constraints. They know that the patient is resistant to other antibiotics such as Cephalosporins, but still, they must prescribe the same salts due to the budget.” RPh-23*

- - 1. ***Inability to Procure - The SML Factor***

The participant stated in the following quote that procuring a few antibiotics was limited due to the restraint imposed by the Standard Medicine List (SML). The participant deemed these antibiotics to be the key to providing optimal treatment. This approach limits healthcare facilities’ local needs and disregards local resistance patterns.

*“If we talk about the DHAs or the whole procurement process, some antibiotics are not even considered. That is, they are not present in the list (SML). (---). For example, ampicillin. It is not part of the list. (---). So, ampicillin is not there and so is linezolid, ceftazidime, and cefotaxime. So, one of the things is that some antibiotics are not available (to procure).” RPh-07*

- - 1. ***Procedural Delays Affecting Supply Chain***

The quote below outlines the challenges affecting the supply chain, encompassing protracted timelines that lengthen the procurement cycle, decision-making bottlenecks, and extended delivery periods due to economic circumstances. These vulnerabilities obstruct the timely supply of medicines to health facilities, thereby upsetting access to medicines.

*“As this is a public procurement process, you can face a lot of undue and, I would say, unseen, unforecasted delays. These may be related to bidding, technical evaluation, or market surveys. So, the timeline is extensive. (---). The second factor is about the decision-makers. This purchase is carried out by committees and notified committees under the rules. (---). So, sometimes, the persons in the committees defer the decisions or make late decisions. Again, this impacts the timeline. The third thing is that the delivery periods may sometimes be extended. This could be due to the country’s current situation (economic) of the country. It affects the availability of raw materials, imports, exports, etc. Again, this will extend the timeline. So, these are the big hurdles in the purchase process.” RPh-12*

Another participant pointed out that the procurement process is subject to attrition, which impairs hospitals' inventory management. Dropouts could pose a challenge to procuring agencies in arranging cost-effective medicines, warranting process optimization to ensure maximum supply coverage in the pooled procurement.

*“As the procurement process moves toward the end, many products have dropped. Suppose you’ve 230 items. Out of this, 120 are quoted, and some will drop out of this.” RPh-10*

- - 1. ***Hospital Pharmacist - An Administrative Role***

Few participants highlighted that they were underperforming as clinical pharmacists due to the shortage of pharmacists and the overburden of managerial and administrative responsibilities. Consequently, missed opportunities prevail in terms of monitoring and auditing prescription trends and drug-resistance patterns. Such circumstances lead to suboptimal quality of care and the inability of pharmacists to develop effective pharmaceutical care plans.

*“These things prevent a clinical pharmacist from doing his job. This is due to the insufficient number of pharmacists. So, in such situations, the pharmacist is stuck in other matters and is unable to provide clinical benefit to the patient he can. He has no spare time to monitor these things such as prescription trends and resistance trends and give his input on antibiotic resistance.” RPh-23*

- - 1. ***Irrational Prescribing Affecting Access to Antibiotics***

Besides budgetary constraints, the unjustified and overprescribing of antibiotics also affects access to antibiotics until the next procurement cycle. The situation explained in the excerpt below is predominantly true for outpatients.

*“It is also due to the lack of budget, but it is also true that our physicians prescribe antibiotics for every condition.” RPh-21*

- - 1. ***Antibiotics - Drugs of Meagre Importance***

In the quote below, the participant indicated budget as a “default problem.” Additionally, the quote below captures a primary issue where the participant emphasized the lack of understanding and due importance given to antibiotics, particularly the potential for antibiotic resistance associated with their inappropriate use, by the relevant authorities. This limited understanding could be the reason for their irrational use and, subsequently, indifferent budgetary allocation for antibiotics.

*“Actually, the biggest problem in the procurement of antibiotics, (---), is the lack of recognition of their importance. As for the budget, this is a problem by default. (---). But the importance of antibiotics is nowhere to be seen among the government, people, or the decision-makers.” RPh-12*

The quote below further strengthens our argument that authorities are inconsiderate towards antibiotics and all the factors related to these drugs.

*“We treat it (antibiotic) as a general drug. We do not take into consideration its resistance risk. It has a negative impact that the antibiotic is treated as an ordinary medicine, like a painkiller or other kind of drug.” RPh-22*

- - 1. ***Unavailability of Susceptibility Testing***

The participants expressed concerns regarding the non-availability of antibiotic susceptibility testing in hospitals. This pervasive limitation hampers the ability of the hospital and, subsequently, the province to collect the resistance profiles of the microbes, thereby preventing the development of a data repository, which ultimately promotes empirical prescribing, leading to irrational utilization of antibiotics, further augmenting antimicrobial resistance (AMR). The quote explicitly highlights the lack of infrastructure for culture sensitivity testing within hospital laboratories. The availability at a cost bearable by the patient will eventually promote clinicians' use of this diagnostic service.

*“So, about the resistance, there is currently no such setup in our hospital or even at the primary and secondary care level (department) to record this data. (---) You have to go to private laboratories and rely on their reports. So, if culture testing is available, it must be affordable as well. Then definitely, the doctors as well as consultants will opt for culture testing.” RPh-07*

Another participant accepts that sensitivity testing is normally not a first-line diagnostic measure; in critical situations where empirical therapy fails, it is performed to devise a further course of treatment. However, to optimize diagnostic stewardship, the timing of the sample taken for culture sensitivity is critical for accurately interpreting the culture sensitivity results.[2]

*“However, it is not ultimately that they will not opt for the sensitivity test in the government hospital and solely rely on empirical therapy. If there is a patient whose fever is not getting aside despite the administration of antibiotics, then, surely, they will opt for this life-saving culture sensitivity test. So, yes! Not only do they rely on their clinical experience but also perform culture and sensitivity tests.” RPh-12*

- - 1. ***Perception Rather Misconception about the Resistance Pattern***

There is a common misconception among prescribers and pharmacists that narrow-spectrum antibiotics like amoxicillin and sulfamethoxazole are among the resistant drugs. Few participants presented this view but did not produce or discuss any evidence to support their claim. Rather, in a few other instances, most participants claimed they were unaware of the local resistance pattern. The reason behind their obliviousness is the sparse utilization of culture sensitivity testing within their facility. So, assuming an antibiotic to be resistant deprives it of being prescribed/consumed, and hence, the antibiotic loses its budgetary space from the forthcoming procurement cycle. This strongly correlates with physicians' prescribing behavior, which requires constant prompting and updated information regarding antibiotics.

- 1. **Recommendations for the Future**
     1. ***Nurturing the Clinical Role of Pharmacist***

The clinical role of pharmacists in secondary care health facilities has not flourished as expected. Overburden with administrative tasks has been reported to be a notable barrier. Few participants admitted to occasional training sessions conducted for clinical pharmacists, which appears inadequate. Few participants suggested a thorough, comprehensive clinical training boot camp at setups where the role of the clinical pharmacist is well-established. The quote below captures a disappointing situation; however, it is necessary to nurture the clinical pharmacy practice at governmental institutions in a true spirit to optimize medication management.

*“The CPPO (Clinical pharmacist and Pharmacovigilance officer) is a good project launch with good intentions, but it did not give the desired outcome despite being a good initiative by the primary and secondary care department. (---). They (CPPO) did not have the required knowledge, expertise, or any connection with the clinical side. They (the department) just issued a letter and notified the CPPOs.”* RPh-09

- - 1. ***Acceptance in the System***

Although many participants have shown discontent regarding accepting the pharmacist as a practicing clinical pharmacist, the comment below explains the situation in a much more rational and unbiased way.

*“The concepts of physicians regarding the pharmacists and their role are also clearing up. Their acceptance is rising, given their elaborated role. But the problem is that it varies from person to person. Since the pharmacist (profession) is booming, it is taking time for acceptance. Now, the opinion of a pharmacist is considered, but the voice is not quite impactful. The call is made, no doubt, but its impact is mild.” RPh-12*

- - 1. ***Clinical Pharmacists: Playing a part in Procurement***

Setting aside contemporary barriers, clinical pharmacists can play an effective role in antibiotic procurement. A participant suggested incorporating the clinical pharmacist’s right in the secretariate, i.e., Lahore, where policy decisions occur. This move could help optimize the procurement process concerning the inclusion/selection of antibiotics. Furthermore, the secretariat could collect and evaluate necessary data regarding the resistance profiles from all institutions and act to revise SML accordingly.

*“They (Clinical Pharmacists) should be installed as a test stone in the procurement process right at the place from where the procurement begins, i.e., in Lahore. Then the requirements should be established by taking into account their advice, e.g., avoiding the things that have a higher potential of resistance.” RPh-09*

- - 1. ***“Real-time (Resistance) Data”***

The participant in the comment below rightly pointed towards opting for the data-driven approach for antibiotic procurement. This could improve the rational use of antibiotics.

*“Certainly, if we are to ensure the rational procurement of antibiotics, the most important thing is the real-time data. Patient data should be available in the hospital facility where you have to procure medicines. Apart from that, we have to see which infectious diseases are very common in our area. Right? Which antibiotic seems to have encountered more resistance in a specific population? So, if antibiotics are procured by taking this into account, as well as the financial constraints and all other factors, then they can be used more rationally.” RPh-01*

- - 1. ***Strengthening Clinical Pharmacists with Data***

Another participant exhibited a similar idea and explained in detail that leveraging clinical pharmacists with the data on resistance patterns could reap beneficial outcomes for antibiotic rationalization. However, data generation is a by-product of culture and sensitivity profiling of patients, which is seldom performed at primary and even secondary-level hospitals. The participant added that creating an electronic data repository of resistance profiles would be a key initiative to tackle the AMR threat.

*“First, some kind of data (of resistance) should be available to the clinical pharmacist. If cultures are not available, how is the pharmacist supposed to know if an infection is resistant or susceptible to Tanzo® (Bosch), piperacillin/tazobactam? (---). Moreover, if we collect this data locally and preserve it through some software or any other mechanism, it would be more useful for the Pharmacist, and he can add his/her input in a much better way.” RPh-07*

- - 1. ***Infrastructural and Budgetary Requirements***

Budgetary grants and infrastructural soundness are the mainstay in the fight against AMR, and these impediments should be addressed appreciably to achieve diagnostic stewardship.

*“Firstly, the infrastructure should be strong enough that if there is a culture test, it rapidly reports the results. If the report is generated timely, it can also reduce the burden of antibiotic use.” RPh-03*

*“Another important thing is that the hospital shall have the resources to carry out the culture and sensitivity tests in the labs. A budget should be allocated for them so that the patients can undergo sensitivity testing rather than just relying on empirical therapy.” RPh-12*

- - 1. ***Implementation of Antibiotic Stewardship Program***

The participants emphasized implementing a stewardship program for optimal patient outcomes.

*“I believe that if we implement the stewardship program in its true sense and administer the antibiotics according to the patient's needs, we can treat them in a better way.” RPh-08*

- - 1. ***Antibiotic Stewardship – The Vertical Program***

Unequivocally, the current status of AMR around the globe warrants serious measures to utilize antibiotics in healthcare settings judiciously. An insightful comment by the participant is presented below: the participant recommended a separate vertical program dealing with AMR, which facilitates stewardship goals, would be a need of the hour.

*“You have to deal with it as an independent section. Just as we run vertical programs in the government sector, we will have to tag it and run it as a separate program – the antimicrobial stewardship program. All purchases should fall under this program. (---). Take the example of TB (tuberculosis). (---). They developed TB clinics throughout Punjab. Similarly, antimicrobial stewardship should be turned into a flagship program.” RPh-12*

- - 1. ***Clinical Audits across the Facilities***

A participant recommended clinical audits by the stewardship team at the district level. A similar arrangement can be made at the facility level where the intrinsic hospital level committee could perform a drug utilization review monthly or quarterly or as suited to optimize antibiotic utilization. A hospital antibiotic stewardship committee should be mandated for this job.

*“Let’s say there are three hospitals in the DHA, the DHQ and THQ’s, they all should have a committee that will further evaluate the antibiotics used in their respective DHA and what are the other antibiotics that are already being used. (---). There should be a notification of committees from the department (---). These committees shall have weekly meetings and set guidelines according to stewardship protocols. The prescription shall also be in accordance with these guidelines. Then there should be teams that will evaluate the physicians or perform an inspection by asking why a different antibiotic has been prescribed while the guidelines mention otherwise. It will also provide some new findings, making it easier to rule out errors in the future.” RPh-04*

- - 1. ***Stewardship Through Procurement***

Antibiotic procurement cannot be delinked from the stewardship program but should be perceived as an important component. Ensuring timely access to antibiotics and keeping the supply chain drought-proof fulfills stewardship goals [3]. The participant eloquently elaborated on the concept that procurement of antibiotics must be carried under the umbrella of an ASP, considering local resistance profiles and hospital utilization patterns.

*“See, the thing is, first of all, we should introduce antimicrobial stewardship programs in the hospitals. It should be a proper committee consisting of personnel from every department. Once this committee is formed, the next step is to have an annual procurement agenda that will develop further guidelines by discussing the demand and utilization of antibiotics. Now, in this scheme, everything will be carried out under the antimicrobial stewardship program. Right? It will give us insights into everything, such as the inflows and outflows of antibiotics, their utilization, cost, and local resistance patterns. This way, we can implement it effectively.” RPh-22*

- - 1. ***Continuous Medical Education***

The quote below emphasizes continuous medical education (CMEs) and highlights its importance, particularly for pharmacists. CMEs should be made a regular part of professional growth, which is ultimately linked to the uplifting of the profession.

*“Moreover, pharmacists, the voice of antimicrobial stewardship in hospitals, should be empowered. They should be enlightened, trained, and given further education. Of course, their degree is valuable, and if they secure more of it through postgraduate endeavors, it would strengthen their voice. People would start listening to them. So, raising awareness about the importance of antibiotics is essential.” RPh-12*

The participant also recommended training sessions for the prescriber so that prescribing is based on the updated evidence and improves the patient’s clinical outcomes.

*“Sir, in this regard, I think there should be training sessions for the doctors to generate the prescription according to the patient's needs.” RPh-23*

- - 1. ***Adhering to WHO’s AWaRe Classification***

The participant’s suggestion to comply with the WHO’s AWaRe Classification to optimize antibiotic consumption is encouraging. SML should be designed to reflect compliance with WHO’s AWaRe through the procurement process.

*“One of the most important targets of WHO’s AWaRe classification is that there should be more than a 60% consumption of antibiotics of the Access group in any primary and secondary care hospital. (---). So, we should design a procurement process so that 60 % of the antibiotics come from the WHO Access group. Right? Then, about 20 to 30% should come from the Watch group so that we can utilize them in severe infections while keeping in view the local resistance data. The remaining 5 to 10% should be reserved for the ICU, where patients suffer from life-threatening situations. If this is followed, I think we can make the process much better.” RPh-22*

- - 1. ***Autonomous Procurement***

Participants presented their arguments regarding the procurement of antibiotics or at least selecting antibiotics per their hospitals' local resistance pattern. Opinions were divided among participants in this regard. Both parties offered valid arguments; however, a mechanism should be established through which the local resistance pattern of health facilities carries weight in the procurement of antibiotics.

*“Yes, (---). I think that each facility should procure according to its budget, local population, and AMR. (---). So, definitely, there should be a localized policy or system that we can follow for our local purchases. Instead of following a list made by the central government,” RPh-22*

*“I think that this will not be right. There would be problems with quality assurance and uniformity. (---). The reason is that when you move to the micro level, people will try to add undue salts and molecules to the list. They can’t control the higher authorities but can use their influence on local authorities.” RPh-12*

**References**

1. Government of Punjab. Punjab Procurement Rules - 2014 Amended Upto 31-12-21 [Internet]. 2014. Available from: https://ppra.punjab.gov.pk/system/files/PPRA%20Rules%20%28Amended%2031-12-2021%29.pdf

2. John G, Mugnier E, Pittet E, Staehli DM, Clerc O, Kenfak AF, et al. Urinary culture sensitivity after a single empirical antibiotic dose for upper or febrile urinary tract infection: A prospective multicentre observational study. Clin Microbiol Infect. 2022;28:1099–104.

3. Malan L, Labuschagne Q, Brechtelsbauer E, Goff DA, Schellack N. Sustainable access to antimicrobials; a missing component to antimicrobial stewardship—a tale of two countries. Front Public Health. 2018;6:324.
