# Supplementary material for: The antibiotic procurement saga: a long-neglected stewardship target to combat antimicrobial resistance in Pakistan
Source: Antimicrob Resist Infect Control. 2025 Feb 7;14:7. doi: 10.1186/s13756-025-01521-w (PMC11806573; doi:10.1186/s13756-025-01521-w)
Supplement: Supplementary file 2 — Supplementary Material 2 [file 13756_2025_1521_MOESM2_ESM.docx]

Interview Guide: **Antibiotic Procurement in Public Sector Hospitals**

**Part 1: Introduction**

- Introduce yourself and introduce the study. Tell what the interview will be used for
- Ask Permission to record the interview on tape
- Assure the anonymity of the interviewee
- Ask the interviewee to introduce themself, including age, education, and years of experience as a Hospital Pharmacist

| **Interviewee Code: Recording Code:** |
| --- |
| **Gender: Male Female** |
| **Age:** |
| **Pharmacy School:** |
| **Year of Graduation:** |
| **Experience in Public Sector (years): Qualification:** |
| **Hospital Bed Strength: Procurement Budget:** |
| **How long have you been taking part in procuring medicines?** |
| **What role do you play as a pharmacist in the procurement of medicines?** |
| **How much do you spend on procuring antibiotics (Bulk + LP)?** |

**Part 1: Basic Knowledge and Attitude**

- From where do you have your basic knowledge about antibiotics, and how do you keep yourself up-to-date?
- What is your general understanding and awareness of the importance of antimicrobial stewardship and its impact on patient care?
- Do you have any ideas about antibiograms?
- Do you know about your hospital's current resistance status? If yes, by which means did you get that information?
- Are you aware of the WHO’s AWaRe Classification of Antibiotics?

**Part 2:** **Understanding of the Procurement Process**

- Can you please describe your role as a pharmacist in your hospital's medicine procurement process?
- Can you walk me through your hospital's current antibiotic procurement process?
- How do you set demand for antibiotics, or which criterion do you follow for setting demand for antibiotics?
- Which factors are currently considered when making procurement decisions for antibiotics?
- Are antibiograms or current resistance trends considered during the procurement process? **If yes**, how are they utilized? **If not**, why do you think they are not considered?
- Is the current procurement process broad and inclusive enough to consider the antimicrobial resistance (AMR) status of the province and, subsequently, the hospital?

**Part 3: Access to Antibiotics in Hospitals**

- In your opinion, does the current procurement process ensure access to antibiotic supply throughout the year or the next procurement cycle?
- Do you think there is timely access to antibiotics for the patients?
- How do you cover antibiotic shortages? If there is one.

**Part 4: Impact of Clinical Pharmacist or Clinical Input from Pharmacist on**

**Antibiotic Procurement** **Decision Making**

- In your opinion, how would involving a clinical pharmacist or obtaining clinical input from pharmacists improve the antibiotic procurement process?
- Do you think including a clinical pharmacist would encourage rational antibiotic procurement?
- Do you think that the involvement of clinical pharmacists will impact antibiotic utilization and antibiotic cost in the hospital?
- Do you think the current procurement process shows enough flexibility to consider clinical input while procuring antibiotics?

**Part 5: Challenges and Limitations**

- What challenges or limitations do you currently face during the procurement process, particularly about antimicrobials?
- Have you faced any specific challenges or limitations when involving clinical pharmacists or clinical input in the procurement decision-making process for antibiotics?

**Part 6: Recommendations for Improving the Procurement Process**

- Based on your experience, what recommendations do you have to make the antibiotic procurement process more effective and efficient, particularly regarding antimicrobials?
- How do you think the involvement of clinical pharmacists can be integrated into the existing procurement process for better outcomes?
- Do you suggest any specific strategies or changes to enhance antimicrobial stewardship through the procurement process?

**Finalizing Interview**

- Thank the interviewee for spending their time with you
- Ask if there is any additional comment they want to make about the questions asked during the interview
- Tell the interviewee what will happen to the recordings now
