# Supplementary material for: The antibiotic procurement saga: a long-neglected stewardship target to combat antimicrobial resistance in Pakistan
Source: Antimicrob Resist Infect Control. 2025 Feb 7;14:7. doi: 10.1186/s13756-025-01521-w (PMC11806573; doi:10.1186/s13756-025-01521-w)
Supplement: Supplementary file 4 — Supplementary Material 4 [file 13756_2025_1521_MOESM4_ESM.pdf]

PURCHASE CELL
DIRECTORATE GENERAL
HEALTH SERVICES PUNJAB
24-COOPER ROAD, LAHORE

Primary & Secondary
Healthcare Department

Phone No. +924299201145 e-mail: pcdghslahore@gmail.com

No. 904-20 Date: 24/01/2024

To,

1. **Chief Executive Officers**
   District Health Authorities Punjab.
2. **Medical Superintendents**
   District Headquarter Hospitals and District Headquarter (Level) Hospitals under administrative control of P&SHD
3. **Medical Superintendents**
   Tehsil Headquarter Hospitals and Tehsil Headquarter (Level) Hospitals under administrative control of P&SHD
4. **SMO's / Incharge, Rural Health Centers**
5. **Incharge, Basic Health Units (24/7)**
6. **Incharge, Basic Health Units**

Subject: **NOTIFICATION OF STANDARD MEDICINE LIST (SML) – 2024 FOR BULK PURCHASE ALONG WITH INDICATIVE QUANTITIES FOR PRIMARY & SECONDARY HEALTHCARE FACILITIES**

Kindly refers to the subject cited above.

2. Directorate General Health Services, Punjab, in suppression of the notification No. 3805-53/PC Dated 03-03-2023, wherein Standard Medicine List (SML) – 2023 was notified, in consultation with all stake holders including Medical Superintendents, Consultants, Senior Medical Officers, Medical Officers, Pharmacy Managers, Technical experts of USAID Global Health Supply Chain – Procurement and Supply Management (GHSC-PSM) Pakistan and procuring agencies of P&SHD has standardized the list of medicines along with indicative quantities to be made available.

3. Keeping in view of the procurement process of Medicine and Medical Devices for FY 2022-23 and FY 2023-24 as well as the suggestions received from multiple health facilities, the Directorate General Health Services, Punjab called upon consultative sessions of stake holders as well as circulation of draft notification dated 15-01-2024, hereby, notify Standard Medicine List (SML) – 2024 with following guidelines.

   i. Individual entity/ Procuring Agency may increase/ decrease quantities of individual item within 30% range at their own level. But any increase shall be adjusted in other less required item(s) by keeping in view of overall budget.
   ii. For increase/ decrease in more than 30%, and addition / deletion of any item is required in the standard medicine list for bulk purchase, matter will be referred to committee headed by DGHS, Punjab who shall approve or reject the same within seven working days. It is advisable that such arrangement may be made at the procurement planning stage.

In case of non-reply, the concerned procuring entity may approach 1033 / Department helpline for early decision.

iii. Overall orders shall be within allocated amount for the year and corresponding prorata adjustment may be made equally to all items to adjust the quantities as per available budget allocation.

iv. The procuring agency will hold fortnight meetings to review the medicines utilization at facilities and will pass directions for equal utilizations of same group of medicines at a time.

v. The items for dialysis ward, Dental, Blood Bank will be dealt separately as per their respective budget allocations.

4. Furthermore, likewise in SML -2023 a Supplementary / Optional list of Medicine and Medical Devices is also included which can be procured after the fulfilment of regular Standard Medicine List – 2024. But overall budget utilization on the Supplementary / Optional list shall not exceed 5% of the total allocated budget for bulk procurement.

5. All the Procuring Agencies under Primary & Secondary Healthcare Department are required to follow this standard list of medicines for bulk purchase along with indicative quantities as a policy decision.

-Sd/-
DIRECTOR GENERAL HEALTH SERVICES
PUNJAB

CC.
No. & Date Even
A copy is forwarded for information and further necessary action to;

1. Secretary Primary & Secondary Healthcare Department, Punjab.
2. Special Secretary (Operations /Development), P&SHCD, Punjab.
3. Additional Secretary Drug Control/Technical/Vertical Programs/Development P&SHCD, Punjab.
4. Director General Drugs Control, P&SHCD, Punjab
5. Deputy Secretary (Tech-II) / Drugs Control, P&SHCD, Punjab
6. Director Health Services (HQ) DGHS.
7. General Manager, Govt. Medical Store Depot, Lahore.
8. Additional Director MS&DC DGHS.
9. Deputy Director, HISDU P&SHCD with the request to upload on departmental website.
10. Pharmacy Managers, DHQ and THQ Hospitals, P&SHD
11. Master File.

DIRECTOR PHARMACY
DIRECTORATE GENERAL HEALTH SERVICES, PUNJAB

Standard Medicine List (SML) - 2024													
**Sr**	**List**	**Generic Name**	**A-DHQ Beds 450+**	**B-DHQ Beds 350-449**	**C-DHQ Beds 250-349**	**D-DHQ Beds 125-249**	**A-THQ Beds 120+**	**B-THQ Beds 90-119**	**C-THQ Beds 41-89**	**D-THQ Beds 20-40**	**RHC**	**BHU 24/7**	**BHU**
1	IPD/ER	Absorbent Cotton Wool 500g Roll	6,000	5,000	3,000	1,500	1,500	1,200	800	400	70	60	30
2	IPD/ER	Acetylsalicylic acid 300mg Tab/ Cap	12,000	10,000	8,000	6,000	8,000	6,000	5,000	3,000	-	-	-
3	IPD/ER	Acyclovir 500mg Infusion	800	600	400	200	-	-	-	-	-	-	-
4	IPD/ER	Amikacin (Sulfate) 250mg Injection	8,000	6,000	4,000	2,000	1,500	1,000	800	600	200	-	-
5	IPD/ER	Aminophylline 25mg/ml Injection	150	100	100	100	100	80	60	40	-	-	-
6	IPD/ER	Amiodarone HCl 150mg Injection	240	180	120	60	40	30	20	10	-	-	-
7	IPD/ER	Amiodarone HCl 200mg Tab/ Cap	600	400	300	200	200	100	50	50	-	-	-
8	IPD/ER	Amoxicillin + Clavulanic Acid 1.2g Injection	6,000	5,000	4,000	3,000	2,000	1,500	1,000	800	-	-	-
9	IPD/ER	Anti-Rabies Vaccine (PVRV) Injection	2,500	2,000	1,500	1,000	1,500	1,200	1,000	800	50	-	-
10	IPD/ER	Anti-Snake venom Serum Injection	200	150	120	100	100	80	60	50	10	-	-
11	IPD/ER	Atracurium (besylate) 10mg/ml Injection	1,000	800	700	600	500	400	300	200	-	-	-
12	IPD/ER	Atropine (Sulfate) 1mg/ml Injection	1,400	1,200	1,000	800	600	500	400	300	50	20	10
13	IPD/ER	Auto Disposable Syringe 3cc	150,000	120,000	100,000	80,000	80,000	60,000	50,000	40,000	8,000	3,000	1,000
14	IPD/ER	Auto Disposable Syringe 5cc	600,000	500,000	400,000	300,000	300,000	200,000	150,000	100,000	10,000	5,000	2,000
15	IPD/ER	Bandage Plaster of Paris BPC, 10cmX2.7m Roll	7,200	5,400	3,600	1,800	1,800	1,200	900	720	-	-	-
16	IPD/ER	Beclomethasone Dipropionate 800mcg Nebulizer Solution	1,000	800	600	400	500	400	300	200	-	-	-
17	IPD/ER	Black Silk Size 1 30mm,1/2 circle	2,100	1,425	1,050	525	480	384	300	204	120	60	30
18	IPD/ER	Black Silk Size 2/0 30mm,1/2 circle	2,088	1,416	1,044	522	480	384	300	204	-	-	-

Sr	List	Generic Name	A-DHQ Beds 450+	B-DHQ Beds 350-449	C-DHQ Beds 250-349	D-DHQ Beds 125-249	A-THQ Beds 120+	B-THQ Beds 90-119	C-THQ Beds 41-89	D-THQ Beds 20-40	RHC	BHU 24/7	BHU
19	IPD/ER	Blood Bags Sterile Packs 500ml single	1,500	1,200	1,000	800	800	600	400	200			
20	IPD/ER	Blood Transfusion Set	1,500	1,200	1,000	800	800	600	400	200	-	-	-
21	IPD/ER	Bupivacaine HCl 0.50% Injection	2,000	1,500	1,200	600	800	600	400	300	-	-	-
22	IPD/ER	Bupivacaine HCl 0.75% Spinal Injection	3,000	2,600	2,200	1,800	1,600	1,400	1,200	1,000	-	-	-
23	IPD/ER	Calcium Gluconate 100mg/ml Injection	800	600	500	400	500	400	300	200	50	-	-
24	IPD/ER	Captopril 25mg Tab/ Cap	20,000	18,000	16,000	14,000	12,000	10,000	8,000	6,000	400	120	60
25	IPD/ER	Catgut Chromic size 1, 30mm	2,376	1,632	1,188	594	480	384	300	204	120	60	30
26	IPD/ER	Catgut Chromic size 2/0, 30mm, 1/2 circle	2,688	2,016	1,344	672	480	384	300	204	-	-	-
27	IPD/ER	Cefoperazone + Sulbactam 2G Injection	6,000	5,000	4,000	3,000	3,000	2,000	1,500	1,000	-	-	-
28	IPD/ER	Ceftriaxone (Sodium) 1G Injection	80,000	65,000	50,000	45,000	35,000	30,000	25,000	20,000	5,000	2,000	-
29	IPD/ER	Ceftriaxone (Sodium) 250mg Injection	20,000	15,000	12,000	10,000	15,000	12,000	10,000	8,000	1,000	200	-
30	IPD/ER	Charcoal (Activated) 250mg Powder	60	45	30	15	20	20	20	20	-	-	-
31	IPD/ER	Ciprofloxacin HCl 200mg/100ml Infusion	5,000	4,000	3,000	2,000	3,000	2,500	2,000	1,500	-	-	-
32	IPD/ER	Cotton Bandage BPC 10cmX6m Roll	3,000	2,500	2,000	1,000	1,000	800	600	400	-	-	-
33	IPD/ER	Cotton Bandage BPC 6.5cmX6m Roll	12,000	10,000	8,000	6,000	5,000	4,000	3,000	2,500	500	360	240
34	IPD/ER	Cotton Crape Bandage 7.5cmX4.5m Roll	3,000	2,500	2,000	1,500	1,500	1,200	1,000	800	-	-	-
35	IPD/ER	Cotton Crepe Bandages 10cmX4.5m Roll	840	630	420	210	200	140	110	70	-	-	-
36	IPD/ER	Desferioxamine 500mg Injection	200	150	100	50	100	80	60	30	-	-	-
37	IPD/ER	Dexamethasone 4mg/ml Injection	40,000	30,000	20,000	16,000	20,000	16,000	12,000	8,000	-	-	-
38	IPD/ER	Dextrose + Saline (1000ml) Infusion	4,000	3,000	2,000	1,500	2,000	1,500	1,200	1,000	-	-	-

Sr	List	Generic Name	A-DHQ Beds 450+	B-DHQ Beds 350-449	C-DHQ Beds 250-349	D-DHQ Beds 125-249	A-THQ Beds 120+	B-THQ Beds 90-119	C-THQ Beds 41-89	D-THQ Beds 20-40	RHC	BHU 24/7	BHU
39	IPD/ER	Dextrose 10% (1000ml) Infusion	3,000	2,500	2,000	1,500	2,000	1,500	1,000	800	-	-	-
40	IPD/ER	Dextrose 25% Injection	6,000	5,600	5,200	4,800	4,800	2,400	3,000	2,000	200	-	-
41	IPD/ER	Dextrose 5% (1000ml) Infusion	2,400	1,800	1,200	600	1,500	1,300	1,100	900	300	200	100
42	IPD/ER	Diazepam 10mg Injection	6,400	4,800	3,200	1,600	900	750	600	450	-	-	-
43	IPD/ER	Diclofenac (Sodium) or Potassium75mg Injection	120,000	100,000	80,000	60,000	60,000	50,000	40,000	30,000	4,000	2,000	1,000
44	IPD/ER	Dimenhydrinate 50mg/ml Injection	5,000	4,000	3,000	2,000	2,500	2,000	1,500	500	500	200	100
45	IPD/ER	Disposable Syringe 10cc	70,000	60,000	40,000	20,000	30,000	20,000	15,000	10,000	3,000	500	-
46	IPD/ER	Disposable Syringe 1cc (Insulin Syringe)	25,000	20,000	15,000	12,000	18,000	15,000	12,000	10,000	-	-	-
47	IPD/ER	Disposable Syringe 20cc	4,000	3,000	2,000	1,000	1,000	800	600	400	-	-	-
48	IPD/ER	Dobutamine HCl 250mg/5ml Injection	960	880	400	700	300	250	200	150	-	-	-
49	IPD/ER	Dopamine HCl 200mg/5ml Injection	880	720	480	480	220	160	110	90	-	-	-
50	IPD/ER	Drotaverine 40mg/2ml Injection	50,000	40,000	35,000	30,000	30,000	25,000	20,000	15,000	2,000	500	150
51	IPD/ER	Endotracheal tube (all sizes)	760	570	380	190	200	142	108	76	-	-	-
52	IPD/ER	Epinephrine HCl/Adrenaline 1mg/ml Injection	1,400	1,200	1,000	800	600	500	400	300	50	20	10
53	IPD/ER	Erythropoietin 4000 IU Injection	100	80	60	40	60	40	30	20	-	-	-
54	IPD/ER	Examination Gloves All Size	54,000	48,000	42,000	36,000	42,000	36,000	30,000	24,000	-	-	-
55	IPD/ER	Foley’s catheter (all sizes)	6,000	5,000	4,000	3,000	3,000	2,500	2,000	1,500	-	-	-
56	IPD/ER	Furosemide 20mg/2ml Injection	50,000	40,000	35,000	30,000	25,000	20,000	15,000	10,000	1,000	-	-
57	IPD/ER	Glyceryl Trinitrate (Sublingual) 0.5mg Tab/ Cap	10,000	8,000	6,000	4,000	4,000	3,000	2,000	1,500	150	60	30
58	IPD/ER	Heparin (Sodium) Injection	1,000	800	600	400	400	300	200	100	-	-	-

Sr	List	Generic Name	A-DHQ Beds 450+	B-DHQ Beds 350-449	C-DHQ Beds 250-349	D-DHQ Beds 125-249	A-THQ Beds 120+	B-THQ Beds 90-119	C-THQ Beds 41-89	D-THQ Beds 20-40	RHC	BHU 24/7	BHU
59	IPD/ER	Hydralazine HCl 20mg/ml Injection	240	180	120	60	40	30	20	20	-	-	-
60	IPD/ER	Hydrocortisone (Sodium succinate) 250mg Injection	20,000	16,000	14,000	12,000	10,000	8,000	6,000	4,000	1,000	500	200
61	IPD/ER	Insulin comp 70/30 Injection	25,000	20,000	15,000	12,000	18,000	15,000	12,000	10,000	-	-	-
62	IPD/ER	Insulin NPH Injection	200	150	100	60	80	60	40	20	-	-	-
63	IPD/ER	Insulin Regular Injection	600	500	400	200	400	300	200	100	-	-	-
64	IPD/ER	Ipratropium Bromide 250mcg Nebulizer Solution	1,000	800	600	400	500	400	300	200	-	-	-
65	IPD/ER	Iron Sucrose 100mg Injection	8,400	6,300	4,200	2,100	2,000	1,500	1,200	1,000	500	-	-
66	IPD/ER	Isoflurane inhalation Gas	400	300	240	160	60	40	30	20	-	-	-
67	IPD/ER	Isosorbide Dinitrate 10mg/10ml Infusion	2,000	1,500	1,000	600	600	500	400	300	-	-	-
68	IPD/ER	IV Canula (DRAP approved) 20G	10,000	7,000	5,000	3,000	4,000	3,000	2,000	1,000	-	-	-
69	IPD/ER	IV Canula (DRAP approved) 22G	80,000	60,000	40,000	20,000	30,000	20,000	15,000	10,000	3,000	500	200
70	IPD/ER	IV Canula (DRAP approved) 24G	30,000	25,000	20,000	10,000	15,000	12,000	10,000	6,000	500	100	-
71	IPD/ER	IV Sets (Sterile)	200,000	150,000	100,000	70,000	70,000	55,000	40,000	30,000	3,000	1,500	1,000
72	IPD/ER	Ketamine 100mg/2ml Injection	240	180	120	60	150	120	100	80	-	-	-
73	IPD/ER	Ketorolac tromethamine 10mg/ml Injection	3,600	2,700	1,800	900	600	500	400	300	-	-	-
74	IPD/ER	Labetalol HCl 50mg/10ml Injection	1,200	900	600	300	1,000	750	500	250	-	-	-
75	IPD/ER	Lignocaine (hydrochloride) 2% Gel	6,400	4,800	2,600	1,900	1,900	1,300	1,000	700	-	-	-
76	IPD/ER	Lignocaine (hydrochloride) 2% Injection	10,000	8,000	6,000	4,000	4,000	3,500	3,000	2,500	1,000	500	100
77	IPD/ER	Lignocaine + Epinephrine 2% Dental Cartridge	720	540	360	180	500	400	200	60	-	-	-

Sr	List	Generic Name	A-DHQ Beds 450+	B-DHQ Beds 350-449	C-DHQ Beds 250-349	D-DHQ Beds 125-249	A-THQ Beds 120+	B-THQ Beds 90-119	C-THQ Beds 41-89	D-THQ Beds 20-40	RHC	BHU 24/7	BHU
78	IPD/ER	Magnesium Sulphate 500mg/ml Injection	300	250	200	150	200	150	100	50	30	20	10
79	IPD/ER	Mannitol (500ml) 20% w/v Infusion	540	405	270	135	300	225	150	120	-	-	-
80	IPD/ER	Mecobalamin 500mcg Injection	5,000	4,000	3,000	2,000	3,000	2,000	1,500	1,000	-	-	-
81	IPD/ER	Metoclopramide (hydrochloride) 10mg Injection	50,000	40,000	35,000	30,000	30,000	25,000	20,000	15,000	-	-	-
82	IPD/ER	Metoprolol 1mg/ml Injection	240	180	120	60	50	40	30	20	-	-	-
83	IPD/ER	Metronidazole 500mg/100ml Infusion	90,000	70,000	45,000	25,000	28,000	23,000	16,000	11,000	2,000	-	-
84	IPD/ER	Midazolam 1mg/ml Injection	2,400	1,800	1,200	600	600	500	400	300	-	-	-
85	IPD/ER	Misoprostol 200mcg Tab/ Cap	30,000	26,000	22,000	18,000	20,000	16,000	12,000	10,000	6,000	3,000	500
86	IPD/ER	Modified Fluid Gelatin 4% Infusion	1,000	800	600	400	500	350	250	160	30	-	-
87	IPD/ER	Moxifloxacin 400mg Injection	2,000	1,600	1,400	1,200	1,000	800	600	400	-	-	-
88	IPD/ER	Nalbuphine HCl 10mg/ml Injection	12,000	10,000	8,000	6,000	4,000	3,000	2,500	2,000	-	-	-
89	IPD/ER	Naloxone 400mcg/ml Injection	480	360	240	120	60	50	40	30	-	-	-
90	IPD/ER	Nasal Cannula Surgical/Disposables	1,500	1,300	1,000	800	600	400	200	100	-	-	-
91	IPD/ER	Nasogastric tube (all sizes)	1,320	990	660	330	350	270	220	160	-	-	-
92	IPD/ER	Nebulizer Kit Surgical/Disposables	1,800	1,350	900	450	500	400	300	200	-	-	-
93	IPD/ER	Nelton Catheter	1,440	1,080	720	360	400	300	200	100	-	-	-
94	IPD/ER	Neostigmine + Glycopyrrolate 2.5+0.5mg/ml Injection	1,200	900	600	300	500	400	300	200	-	-	-
95	IPD/ER	Norepinephrine 1mg/ml Injection	2,000	1,600	1,200	600	100	80	60	40	-	-	-
96	IPD/ER	Normal Saline 0.9% 1000ml Infusion	40,000	35,000	30,000	20,000	20,000	15,000	10,000	6,000	1,000	400	100

Sr	List	Generic Name	A-DHQ Beds 450+	B-DHQ Beds 350-449	C-DHQ Beds 250-349	D-DHQ Beds 125-249	A-THQ Beds 120+	B-THQ Beds 90-119	C-THQ Beds 41-89	D-THQ Beds 20-40	RHC	BHU 24/7	BHU
97	IPD/ER	Normal Saline 100 ml Infusion	14,000	11,000	8,000	4,000	5,000	3,500	2,500	1,500	300	150	-
98	IPD/ER	Octreotide 0.1mg Injection	600	500	400	200	60	40	30	10	-	-	-
99	IPD/ER	Omeprazole 40mg Injection	40,000	35,000	30,000	25,000	30,000	25,000	20,000	15,000	300	-	-
100	IPD/ER	Ondansetron 2mg/ml Injection	1,000	750	500	400	300	200	150	100	-	-	-
101	IPD/ER	Oxytocin 5IU/ml Injection	10,000	8,000	6,000	4,000	4,000	3,500	3,000	2,500	3,000	2,000	500
102	IPD/ER	Paracetamol 1g/100ml Infusion	20,000	15,000	10,000	5,000	7,000	6,000	5,000	4,000	-	-	-
103	IPD/ER	Peads Saline Solution Injection	12,000	9,000	6,000	3,000	4,500	3,000	2,000	1,500	-	-	-
104	IPD/ER	Pheniramine (maleate) 22.7mg/2ml Injection	18,000	13,500	9,000	4,500	4,000	3,300	2,600	1,900	500	200	100
105	IPD/ER	Phenytoin (sodium) 50mg/ml Injection	480	360	240	120	-	-	-	-	-	-	-
106	IPD/ER	Poly propylene 2/0, 30mm, 1/2 circle RB	1,920	1,440	960	480	480	336	240	144	-	-	-
107	IPD/ER	Poly propylene 2/0, 60mm, 1/2 straight cutting needle	300	225	150	72	96	72	36	24	-	-	-
108	IPD/ER	Poly propylene Size 1, 40mm circle RB Needle	1,440	1,080	720	360	360	240	180	120	-	-	-
109	IPD/ER	Polyglactin size 1, 1/2 circle RB	1,440	1,080	720	360	432	384	144	96	-	-	-
110	IPD/ER	Polyglactin, 2/0, 1/2 circle RB	360	270	180	90	108	96	36	24	-	-	-
111	IPD/ER	Potassium Chloride 7.46% Injection	600	500	300	150	200	150	100	80	40	-	-
112	IPD/ER	Povidone – iodine 10% w/v Solution	3,000	2,800	2,600	2,200	2,600	2,200	1,800	1,600	50	30	20
113	IPD/ER	Povidone – iodine 7.5% w/v Scrub	1,200	900	600	300	400	300	200	100	-	-	-
114	IPD/ER	Pralidoxime 200mg/10ml Injection	60	45	30	15	10	10	10	10	-	-	-
115	IPD/ER	Propofol 200mg/20ml Injection	700	540	360	180	100	80	60	50	-	-	-

Sr	List	Generic Name	A-DHQ Beds 450+	B-DHQ Beds 350-449	C-DHQ Beds 250-349	D-DHQ Beds 125-249	A-THQ Beds 120+	B-THQ Beds 90-119	C-THQ Beds 41-89	D-THQ Beds 20-40	RHC	BHU 24/7	BHU
116	IPD/ER	Protamine Sulphate 100mg/ml Injection	60	45	30	15	10	10	10	10	-	-	-
117	IPD/ER	Ringer's Lactate (1000ml) Infusion	40,000	35,000	25,000	20,000	30,000	25,000	20,000	15,000	1,000	400	100
118	IPD/ER	Salbutamol (Sulfate) 5mg/5ml Solution	480	400	320	240	320	240	200	160	-	-	-
119	IPD/ER	Scalp Vein Set	480	360	240	120	100	80	60	40	-	-	-
120	IPD/ER	Silver Sulphadiazine 1% Cream	2,000	1,500	1,400	1,200	1,000	800	600	500	-	-	-
121	IPD/ER	Sodium Bicarbonate (50ml)1.4% isotonic Injection	840	630	420	210	150	110	90	60	40	-	-
122	IPD/ER	Sodium Phosphate (Enema) Solution	1,000	800	700	600	500	400	300	200	50	20	-
123	IPD/ER	Sodium Valproate 500mg Injection	3,600	2,700	1,800	900	300	250	200	100	-	-	-
124	IPD/ER	Spinal Needle all sizes	3,600	2,700	1,800	900	500	400	300	200	-	-	-
125	IPD/ER	Sterile Gauze Piece	12,000	9,000	6,000	3,000	1,000	800	600	400	360	200	100
126	IPD/ER	Sterile Gauze Roll 1 x 30 m	720	540	360	180	400	300	200	100	-	-	-
127	IPD/ER	Sterile Surgical Gloves 6.5,7.0, 7.5	10,000	8,000	6,000	4,000	6,000	4,500	3,000	2,000	-	-	-
128	IPD/ER	Sterilized Cord Clamps	6,000	5,000	4,000	3,000	4,000	3,000	2,000	1,000	1,000	500	200
129	IPD/ER	Sterilized Surgical Blades all sizes	9,600	7,200	4,800	2,400	2,400	2,000	1,600	1,200	300	200	-
130	IPD/ER	Streptokinase 1.5 million IU Injection	400	200	150	80	50	30	20	10	-	-	-
131	IPD/ER	Suction Catheter	360	270	180	90	96	72	48	36	-	-	-
132	IPD/ER	Surgical Hypoallergenic Paper Tape 1/2"	16,000	14,000	12,000	10,000	8,000	6,000	4,000	3,000	500	200	100
133	IPD/ER	Suxamethonium (chloride) 50mg/ml Injection	240	180	120	60	50	40	30	20	-	-	-
134	IPD/ER	Tazobactum +Piperacillin 500mg+4g Injection	4,000	3,000	2,000	1,000	1,000	800	500	-	-	-	-
135	IPD/ER	Tetanus Toxoid Injection	20,000	18,000	16,000	12,000	16,000	14,000	12,000	10,000	500	200	100
136	IPD/ER	Three way stopper	960	720	480	240	180	150	120	84	-	-	-

Sr	List	Generic Name	A-DHQ Beds 450+	B-DHQ Beds 350-449	C-DHQ Beds 250-349	D-DHQ Beds 125-249	A-THQ Beds 120+	B-THQ Beds 90-119	C-THQ Beds 41-89	D-THQ Beds 20-40	RHC	BHU 24/7	BHU
137	IPD/ER	Tramadol HCl 100mg/2ml Injection	15,000	12,500	10,000	8,500	10,000	8,000	6,000	5,000	-	-	-
138	IPD/ER	Tranexamic Acid 500mg/5ml Injection	14,000	12,000	10,000	8,000	6,000	4,000	3,000	2,000	500	100	50
139	IPD/ER	Urine Bags adult/pads'	6,000	5,000	4,000	3,000	3,000	2,500	2,000	1,500	-	-	-
140	IPD/ER	Vancomycin (HCl) 1g Injection	4,000	3,200	2,400	2,000	2,000	1,500	1,000	800	-	-	-
141	IPD/ER	Vitamin K1 10mg Injection	2,000	1,800	1,600	1,400	1,200	1,000	800	600	100	50	30
142	IPD/ER	Volumetric Chamber (I.V Burette)100ml size	4,000	3,000	2,500	1,500	1,000	800	600	400	-	-	-
143	OPD	Acetylsalicylic acid EC 75mg Tab/ Cap	200,000	160,000	120,000	80,000	96,000	80,000	65,000	50,000	15,000	3,500	2,000
144	OPD	Albendazole 200mg Tab/ Cap	3,000	2,400	1,200	1,200	2,000	1,000	1,000	1,000	800	500	200
145	OPD	Albendazole 200mg/5ml Syp/ Susp	12,000	6,000	2,400	2,400	6,000	6,000	3,000	2,500	900	300	200
146	OPD	Allopurinol 300mg Tab/ Cap	42,000	40,000	30,000	20,000	20,000	15,000	10,000	7,000	-	-	-
147	OPD	Alprazolam 0.5mg Tablet	25,000	20,000	15,000	10,000	10,000	8,000	6,000	4,000	-	-	-
148	OPD	Aluminium Hydroxide + Magnesium Trisilicate/Hydroxide + Simethicone Syp/ Susp	25,000	20,000	15,000	12,000	15,000	12,000	10,000	8,000	2,000	1,500	1,000
149	OPD	Amlodipine 5mg Tab/ Cap	500,000	450,000	400,000	300,000	300,000	250,000	200,000	150,000	75,000	7,500	7,500
150	OPD	Ammonium Chloride + Diphenhydramine + others Syp/ Susp	25,000	20,000	15,000	12,000	15,000	12,000	10,000	8,000	2,000	1,500	1,000
151	OPD	Amoxicillin (trihydrate) 250mg/5ml Syp/ Susp	30,000	22,000	18,000	14,000	10,000	8,000	7,000	6,000	4,000	2,000	2,000
152	OPD	Amoxicillin (trihydrate) 500mg Tab/ Cap	-	-	-	-	-	-	-	-	30,000	20,000	10,000
153	OPD	Amoxicillin + Clavulanic Acid 312mg/5ml Syp/ Susp	30,000	25,000	20,000	15,000	15,000	12,000	10,000	8,000	-	-	-
154	OPD	Amoxicillin + Clavulanic Acid 625mg Tab/ Cap	250,000	220,000	180,000	150,000	150,000	120,000	90,000	60,000	-	-	-

Sr	List	Generic Name	A-DHQ Beds 450+	B-DHQ Beds 350-449	C-DHQ Beds 250-349	D-DHQ Beds 125-249	A-THQ Beds 120+	B-THQ Beds 90-119	C-THQ Beds 41-89	D-THQ Beds 20-40	RHC	BHU 24/7	BHU
155	OPD	Artemether + Lumefantrine 15+90mg Syp/ Susp	2,000	1,800	1,600	1,000	1,000	800	600	300	-	-	-
156	OPD	Artemether + Lumefantrine 20+120mg Tab/ Cap	6,000	4,800	4,000	3,600	3,600	2,400	1,800	1,200	-	-	-
157	OPD	Atenolol 50mg Tab/ Cap	400,000	360,000	320,000	240,000	240,000	200,000	160,000	120,000	-	-	-
158	OPD	Atorvastatin 20mg Tab/ Cap	300,000	250,000	200,000	150,000	200,000	150,000	120,000	100,000	-	-	-
159	OPD	Azithromycin 200mg/5ml Syp/ Susp	5,000	4,000	3,000	2,500	2,500	2,000	1,500	1,200	-	-	-
160	OPD	Azithromycin 500mg Tab/ Cap	150,000	130,000	100,000	60,000	80,000	65,000	50,000	35,000	-	-	-
161	OPD	Betamethasone + Gentamycin Crearm	5,000	4,500	4,000	3,500	3,000	2,500	2,000	1,500	1,000	500	300
162	OPD	Bromazepam 3mg Tab/ Cap	180,000	144,000	108,000	72,000	108,000	72,000	54,000	36,000	-	-	-
163	OPD	Calcium Carbonate 400mg + Vitamin D Tab/ Cap	250,000	150,000	100,000	70,000	200,000	150,000	100,000	60,000	-	-	-
164	OPD	Carbamazepine 100mg/5ml Syp/ Susp	4,800	3,800	2,800	1,800	-	-	-	-	-	-	-
165	OPD	Carbamazepine 200mg Tab/ Cap	96,000	80,000	60,000	40,000	48,000	40,000	30,000	20,000	-	-	-
166	OPD	Carvedilol 6.25mg Tab/ Cap	50,000	40,000	30,000	20,000	-	-	-	-	-	-	-
167	OPD	Cefixime 200mg/5ml Syp/ Susp	15,000	12,000	10,000	8,000	10,000	8,000	6,000	5,000	1,000	-	-
168	OPD	Cefixime 400mg Tab/ Cap	150,000	120,000	100,000	80,000	100,000	75,000	50,000	35,000	4,000	-	-
169	OPD	Cetirizine 10mg Tab/ Cap	240,000	180,000	140,000	100,000	140,000	100,000	70,000	45,000	6,000	4,000	4,000
170	OPD	Cetirizine 5mg/5ml Syrup/Susp	20,000	18,000	16,000	14,000	14,000	10,000	7,000	5,000	2,000	1,000	1,000
171	OPD	Ciprofloxacin + Dexamethasone 0.3% + 0.1% E/Drop	3,600	3,000	2,000	1,500	2,000	1,500	1,000	800	400	200	100
172	OPD	Ciprofloxacin HCl 500mg Tab/ Cap	520,000	460,000	400,000	300,000	240,000	200,000	160,000	120,000	25,000	10,000	8,000

Sr	List	Generic Name	A-DHQ Beds 450+	B-DHQ Beds 350-449	C-DHQ Beds 250-349	D-DHQ Beds 125-249	A-THQ Beds 120+	B-THQ Beds 90-119	C-THQ Beds 41-89	D-THQ Beds 20-40	RHC	BHU 24/7	BHU
173	OPD	Clopidogrel 75mg Tab/ Cap	300,000	300,000	250,000	200,000	200,000	250,000	200,000	150,000	-	-	-
174	OPD	Clotrimazole 10%w/w Vaginal Cream with applicator	2,000	1,800	1,500	1,200	1,500	1,200	900	600	300	100	80
175	OPD	Co-trimoxazole (160/800mg) Tab/ Cap	-	-	-	-	-	-	-	-	6,000	2,500	1,500
176	OPD	Co-trimoxazole (80/400mg) Syp/ Susp	-	-	-	-	-	-	-	-	800	600	400
177	OPD	Dextromethorphan + Pseudoephedrine + others Syp/ Susp	15,000	10,000	12,000	8,000	12,000	8,000	5,000	4,000	-	-	-
178	OPD	Diclofenac (Sodium) 50mg Tab/ Cap	450,000	390,000	330,000	300,000	330,000	270,000	240,000	180,000	15,000	10,000	7,500
179	OPD	Digoxin 250mcg Tab/ Cap	4,000	3,000	2,500	1,500	-	-	-	-	-	-	-
180	OPD	Dimenhydrinate 12.5mg/4ml Syp/ Susp	8,000	7,000	6,000	5,000	4,000	3,000	2,000	1,500	1,000	500	200
181	OPD	Dimenhydrinate 50mg Tab/ Cap	120,000	60,000	72,000	24,000	72,000	48,000	36,000	24,000	2,000	1,000	800
182	OPD	Divalproex sodium 500mg Tab/ Cap	48,000	40,000	30,000	20,000	25,000	20,000	15,000	10,000	-	-	-
183	OPD	Domperidone 10mg Tab/ Cap	120,000	96,000	72,000	48,000	72,000	48,000	36,000	24,000	-	-	-
184	OPD	Domperidone 5mg/5ml Syp/ Susp	20,000	15,000	12,000	6,000	12,000	8,000	6,000	4,000	-	-	-
185	OPD	Doxycycline (hyclate) 100mg Tablet	100,000	80,000	60,000	50,000	80,000	60,000	50,000	40,000	6,000	-	-
186	OPD	Drotaverine 40mg Tab/ Cap	120,000	96,000	72,000	45,000	72,000	45,000	35,000	20,000	5,000	2,000	1,000
187	OPD	Empagliflozin 10mg Tab/ Cap	30,000	20,000	15,000	10,000	15,000	12,000	10,000	8,000	-	-	-
188	OPD	Escitalopram 10mg Tab/ Cap	50,000	40,000	18,000	14,000	20,000	18,000	15,000	10,000	-	-	-
189	OPD	Ferrous salt + Folic Acid Tab/ Cap	400,000	350,000	300,000	250,000	300,000	250,000	200,000	150,000	50,000	40,000	20,000
190	OPD	Ferrous salt + Vitamin B complex Syp/Susp	5,000	4,500	4,000	3,500	3,000	2,500	2,000	1,500	1,000	500	300

Sr	List	Generic Name	A-DHQ Beds 450+	B-DHQ Beds 350-449	C-DHQ Beds 250-349	D-DHQ Beds 125-249	A-THQ Beds 120+	B-THQ Beds 90-119	C-THQ Beds 41-89	D-THQ Beds 20-40	RHC	BHU 24/7	BHU
191	OPD	Fluconazole 150mg Tab/ Cap	3,000	2,500	2,000	1,500	2,000	1,500	1,200	1,000	-	-	-
192	OPD	Flurbiprofen 100mg Tab/ Cap	36,000	28,000	20,000	15,000	15,000	12,000	10,000	8,000	5,000	-	-
193	OPD	Furosemide 40mg Tab/ Cap	48,000	48,000	24,000	12,000	30,000	25,000	20,000	15,000	3,000	-	-
194	OPD	Glimepiride 2mg Tab/ Cap	240,000	180,000	96,000	84,000	100,000	70,000	60,000	42,000	-	-	-
195	OPD	Glyceryl Trinitrate (S.R) 2.6mg Tab/ Cap	50,000	40,000	30,000	240,000	30,000	20,000	15,000	10,000	-	-	-
196	OPD	Hydrocortisone 1% Cream	10,000	8,000	6,000	4,000	6,000	4,000	3,000	2,000	500	200	100
197	OPD	Ibuprofen 100mg/5ml Syp/ Susp	30,000	25,000	15,000	10,000	15,000	10,000	7,000	5,000	5,000	3,000	2,000
198	OPD	Ibuprofen 400mg Tab/ Cap	180,000	140,000	100,000	70,000	100,000	70,000	50,000	35,000	-	-	-
199	OPD	Lactulose 3.35gm/5ml Syp/ Susp	6,000	5,000	4,000	3,000	4,000	3,000	2,500	2,000	-	-	-
200	OPD	Lisinopril 10mg Tab/ Cap	400,000	300,000	240,000	200,000	160,000	120,000	100,000	60,000	20,000	5,000	3,000
201	OPD	Losartan Potassium 50mg Tab/ Cap	400,000	300,000	240,000	200,000	160,000	120,000	100,000	60,000	-	-	-
202	OPD	Mefenamic acid 500mg Tab/ Cap	150,000	100,000	85,000	60,000	100,000	85,000	45,000	30,000	-	-	-
203	OPD	Metformin (hydrochloride) 500mg Tab/ Cap	500,000	400,000	360,000	300,000	300,000	260,000	240,000	220,000	30,000	10,000	5,000
204	OPD	Methyldopa 250mg Tab/ Cap	21,000	7,500	6,000	5,000	6,000	5,000	4,000	2,500	2,000	1,000	500
205	OPD	Metronidazole (benzoate) 200mg/5ml Syp/ Susp	15,000	12,000	9,000	8,000	9,000	6,000	4,500	3,000	3,000	2,000	1,500
206	OPD	Metronidazole 400mg Tab/ Cap	400,000	350,000	300,000	240,000	250,000	200,000	150,000	100,000	20,000	5,000	3,000
207	OPD	Montelukast 10mg Tab/ Cap	150,000	125,000	100,000	70,000	100,000	60,000	50,000	30,000	10,000	1,000	500
208	OPD	Moxifloxacin 0.5% E/Drop	3,500	3,000	2,500	1,500	1,200	1,000	900	600	-	-	-
209	OPD	Multivitamin (Vitamin A, D, E + B Complex etc.) Tablet / Capsule	250,000	150,000	100,000	70,000	200,000	150,000	100,000	60,000	-	-	-

Sr	List	Generic Name	A-DHQ Beds 450+	B-DHQ Beds 350-449	C-DHQ Beds 250-349	D-DHQ Beds 125-249	A-THQ Beds 120+	B-THQ Beds 90-119	C-THQ Beds 41-89	D-THQ Beds 20-40	RHC	BHU 24/7	BHU
210	OPD	Nystatin 100,000 IU/ml Solution	2,400	1,800	1,080	1,080	1,080	1,000	540	360	200	150	100
211	OPD	Omeprazole 20mg Tab/ Cap	500,000	400,000	350,000	300,000	350,000	300,000	250,000	200,000	20,000	8,000	4,000
212	OPD	ORS (WHO RECOMENDED) Powder	36,000	25,000	20,000	10,000	20,000	10,000	12,000	5,000	1,500	1,000	800
213	OPD	Paracetamol 120mg/5ml Syp/ Susp	30,000	25,000	15,000	10,000	15,000	10,000	7,000	5,000	5,000	3,000	2,000
214	OPD	Paracetamol 500mg Tab/ Cap	800,000	600,000	400,000	300,000	300,000	240,000	180,000	120,000	100,000	60,000	40,000
215	OPD	Permethrin 5% lotion	10,000	8,000	6,000	4,000	6,000	4,000	3,000	2,000	1,500	1,000	500
216	OPD	Polymyxin B (Sulphate) + Bacitracin Zinc Eye Ointment	3,500	3,000	2,500	1,500	2,500	2,000	1,500	1,200	400	200	100
217	OPD	Polymyxin B (Sulphate) + Bacitracin Zinc skin Ointment	12,000	10,000	7,500	6,000	7,000	5,000	4,000	3,000	600	300	200
218	OPD	Prednisolone 5mg Tab/ Cap	20,000	16,000	14,000	12,000	14,000	12,000	10,000	8,000	-	-	-
219	OPD	Salbutamol (Sulfate) 100mcg Inhaler	4,000	3,000	2,000	1,500	1,500	1,200	1,000	800	-	-	-
220	OPD	Salbutamol (Sulfate) 2mg/5ml Syp/ Susp	5,000	3,000	2,500	1,500	1,500	1,250	1,000	750	500	250	200
221	OPD	Sitagliptin 50mg Tab/ Cap	80,000	60,000	40,000	30,000	40,000	30,000	25,000	20,000	-	-	-
222	OPD	Sodium Picosulfate 5mg Tab/ Cap	50,000	40,000	30,000	25,000	30,000	25,000	20,000	15,000	1,000	500	300
223	OPD	Spironolactone 100mg Tab/ Cap	48,000	36,000	30,000	20,000	30,000	20,000	15,000	10,000	-	-	-
224	OPD	Tamsulosin HCl 0.4mg Tab/ Cap	25,000	20,000	15,000	10,000	15,000	12,000	10,000	8,000	-	-	-
225	OPD	Terbinafine 250mg Tab/Cap	2,500	2,000	1,500	1,000	-	-	-	-	-	-	-
226	OPD	Theophylline 300mg Tab/ Cap	18,000	15,000	12,000	6,000	10,000	8,000	6,000	5,000	-	-	-
227	OPD	Thyroxine 50mcg Tab/ Cap	30,000	25,000	20,000	15,000	20,000	15,000	12,500	10,000	-	-	-

Sr	List	Generic Name	A-DHQ Beds 450+	B-DHQ Beds 350-449	C-DHQ Beds 250-349	D-DHQ Beds 125-249	A-THQ Beds 120+	B-THQ Beds 90-119	C-THQ Beds 41-89	D-THQ Beds 20-40	RHC	BHU 24/7	BHU
228	OPD	Tobramycin + Dexamethasone 0.3% E/Drop	6,000	4,800	3,600	2,400	3,600	2,400	1,800	1,200	800	400	200
229	OPD	Tranexamic Acid 500mg Tab/ Cap	9,000	6,000	5,000	4,000	7,500	6,000	5,000	3,000	1,000	250	250
230	OPD	Zinc Sulphate 20mg/5ml Syp/ Susp	12,000	8,000	7,000	3,500	7,000	5,000	3,000	2,500	1,500	1,000	500

Supplementary / Optional List

Sr	Name of Item	Sr	Name of Item
1	Amoxicillin (trihydrate) Dispersible tablets 500mg	23	Moxifloxacin 400mg Injection
2	Anti D Immunoglobin Injection	24	Multi Micronutrient Powder
3	Betahistidine 8mg Tablet	25	Nifedipine 10mg Capsule
4	Calcium Acetate 667mg Tablet	26	Oxygen Mask
5	Cetirizine 5mg/5ml Syrup	27	Phenobarbital (sodium) 200mg/2ml Injection
6	Chlorpheniramine Suspension	28	Phenylephrine 10mg/ml Injection
7	Chlorpheniramine maleate 10mg/ml Injection	29	Polymyxin B + Lignocaine Ear Drops
8	Chlorpheniramine maleate 4mg Tablet	30	Pregablin 75mg Capsule
9	Combine Oral Contraceptives Pills (21 Tabs Levonorgestrel and ethinyl estradiol and 7 Tab Ferrous Fumarate	31	Propranolol 10mg Tablet
10	Disposable Syringe 60cc	32	Prostaglandin E2 Vaginal Tablet
11	Emergency Contraceptive Pills (ECP) Levonorgestrel: 1.5mg.	33	Ready to Use Supplementary Food (RUSF)
12	Fixomal roll Surgical/Disposables	34	Ready to Use Therapeutic Food (RUTF)
13	Fusidic Acid + Hydrocortisone Cream	35	Resomal
14	Gentamycin 80mg/2ml Injection	36	Ringer’s Lactate (500ml) Infusion
15	Hydrochlorothiazide 25mg Tablet	37	Serratiopeptidase Tablet
16	Implant Levonorgestrel 75mg implants. (Two Rod).	38	Tab. Mebendazole 500mg Chewable
17	IUCD (Cu-T 380A)	39	Terbinafine HCl 1% w/w Cream
18	IV Canula (DRAP approved) 18G	40	Terbinafine HCl 250mg Tablet
19	Latex Condoms	41	Therapeutic Milk F-100
20	Lincomycin 600mg Capsule	42	Therapeutic Milk F-75
21	Medroxyprogesterone acetate 150mg/ml injection.	43	Tizanidine 2mg Tablet
22	Meropenem 500mg Injection	44	Zinc Sulphate Dispersible Tablet 20 mg

Note: Item mentioned in the above supplementary list can be procured after covering all items in SML-2024 but in no case budget of the supplementary list shall exceed 5% of the total allocated budget
